# Efficacy and safety of Gegen Qinlian decoction in the treatment of type II diabetes mellitus: a systematic review and meta-analysis of randomized clinical trials

**DOI:** 10.3389/fendo.2023.1316269

**Published:** 2024-01-26

**Authors:** YiMei Tan, ShuangHua Liu, MengHe Huang, Hui Cheng, BinBin Xu, HongSheng Luo, QiZhi Tang

**Affiliations:** ^1^ Affiliated Guangdong Hospital of Integrated Traditional Chinese and Western Medicine of Guangzhou University of Chinese Medicine, Foshan, Guangdong, China; ^2^ Jinan University, Guangzhou, Guangdong, China; ^3^ Guangdong Provincial Hospital of Integrated Traditional Chinese and Western Medicine, Foshan, Guangdong, China

**Keywords:** Gegen Qinlian decoction, type 2 diabetes mellitus, traditional Chinese medicine, adverse effect, meta-analysis

## Abstract

**Aim:**

The study aims to systematically assess the efficacy and safety of Gegen Qinlian decoction in the treatment of type 2 diabetes mellitus.

**Methods:**

We systematically searched a total of nine databases from the time of creation to 20 March 2023. The quality of the literature was assessed using the risk of bias assessment tool in the Cochrane Handbook. RevMan 5. 3 and Stata 14.0 were applied to conduct meta-analysis.

**Results:**

A total of 17 studies, encompassing 1,476 patients, were included in the study. Gegen Qinlian decoction combined with conventional treatment was found to significantly reduce FBG (MD = −0.69 mmol/L, 95% CI −0.84 to −0.55, p < 0.01; I^2^ = 67%, p<0.01), 2hPG (MD = −0.97 mmol/L, 95% CI −1.13 to −0.81, p < 0.01; I^2^ = 37%, p=0.09), HbA1c (MD = −0.65%, 95% CI −0.78 to −0.53, p < 0.01; I^2^ = 71%, p<0.01), TC (MD = −0.51 mmol/L, 95% CI −0.62 to −0.41, p < 0.01; I^2^ = 45%, p=0.09), TG (MD = −0.17mmol/L, 95% CI −0.29 to −0.05, p < 0.01; I^2^ = 78%, p<0.01), LDL-C (MD = −0.38mmol/L, 95% CI −0.53 to −0.23, p < 0.01; I^2^ = 87%, p<0.01), HOMA-IR (SMD = −1.43, 95% CI −2.32 to −0.54, p < 0.01; I^2^ = 94%, p<0.01), and improved HDL-C (MD = 0.13 mmol/L, 95% CI 0.09–0.17, p < 0.01; I^2^ = 30%, p=0.24). Only three studies explored the differences in efficacy between GQD alone and conventional treatment in improving glucose–lipid metabolism and insulin resistance, and some of the outcome indicators, such as 2hPG and HDL-C, were examined in only one study. Therefore, the effect of GQD alone on glucose–lipid metabolism and insulin resistance cannot be fully determined, and more high-quality studies are needed to verify it. Publication bias analysis revealed no bias in the included studies.

**Conclusion:**

Gegen Qinlian Decoction has certain efficacy and safety in enhancing glycolipid metabolism and alleviating insulin resistance, potentially serving as a complementary therapy for type 2 diabetes mellitus. Rigorous, large-sample, multicenter RCTs are needed to verify this.

**Systematic review registration:**

https://www.crd.york.ac.uk/prospero/display_record.php?ID=CRD42023413758, PROSPERO CRD42023413758.

## 1 Introduction

Diabetes mellitus is a burgeoning global public health concern. According to the 10th edition of the IDF Atlas, it is projected that by 2045, approximately 783 million individuals worldwide (approximately 12.2% of the global population) will be affected by diabetes, with type 2 diabetes mellitus (T2DM) accounting for over 95% of cases (1). China has the largest number of diabetes patients in the world, accounting for more than a quarter of the world’s population, and the overall prevalence of T2DM in China for the period 2015–2019 has reached 14.92%, according to the Diabetes Map of China released in 2022 (2). T2DM is characterized by progressive b-cell insulin secretion loss, chronic hyperglycemia accompanied by insulin resistance, and metabolic syndrome. It is intricately linked to genetic factors, inflammation, and metabolic stress (3). The disease’s advancement leads to detrimental impacts on vital organs, including the kidneys, heart, retina, blood vessels, and nerves, often culminating in organ dysfunction or even mortality (4–6). In 2021, an estimated 6.7 million adults (aged 20–79) succumbed to T2DM or its complications, constituting 12.2% of all deaths in this age group (7, 8). Globally, 9% of health expenditure is spent on diabetes, amounting to $966 billion (1, 9). Current treatments for T2DM include lifestyle modifications, weight loss, glycemic control, lipid lowering, and microcirculation enhancement (3, 8). However, existing hypoglycemic drugs such as biguanides, thiazolidinediones, glinides, alpha-glucosidase inhibitors, dipeptidyl peptidase IV inhibitors, sodium-glucose cotransporter protein 2 inhibitors, and glucagon-like peptide 1 receptor agonists carry the potential for adverse effects, including gastrointestinal reactions, vitamin B12 deficiency, genitourinary tract infections, hypoglycemia, and liver and kidney impairment (10, 11). Even with good glycemic control, the presence of metabolic memory still makes it difficult to effectively prevent the emergence and progression of T2DM and its complications (12, 13). Therefore, there is a pressing need to identify safer and more effective treatments.

It has been shown in numerous studies that Chinese herbs have antioxidant activity, regulating the intestinal flora, alleviating insulin resistance, and protecting pancreatic islet function through multiple pathways, so as to significantly improve the clinical symptoms and quality of life of patients with T2DM, reduce the incidence of adverse effects, and consolidate the clinical efficacy (14–17). For example, berberine, the active ingredient in Huanglian, can improve insulin resistance in target tissues, thus exerting a blood glucose-lowering effect (14). Pueraria Mirifica, the active ingredient of Pueraria Mirifica, can play a role in lowering glucose by increasing insulin sensitivity and regulating glucose and lipid metabolism (15). Baicalin, the active ingredient of Scutellaria baicalensis, exerts hypoglycemic effects by inhibiting gluconeogenesis (16). Pan Jingqiang examined the effect of GQD on glucose tolerance in model animals through animal experiments and found that it has the hypoglycemic effect of sulfonylureas and has antioxidant activity (17).

Gegen Qinlian Decoction (GQD) is derived from the classical work “Treatise on Miscellaneous Diseases of the Typhoid Fever”, comprising Pueraria Mirifica, Scutellaria Baicalensis, Rhizoma Coptidis, and Licorice. According to the original formula, the ratio between the four drugs is 8:3:3:2, but the current clinical utilization is mostly based on the patient’s clinical performance to add or subtract the dosage of the corresponding drugs. Tong Xiaolin divided patients into high-, medium-, and low-dose groups (120 g, 72g, and 24g) through a multi-phase clinical trial and finally found that each dose group could control blood glucose to a certain extent (18, 19). In a study by Zhang Jiacheng, it was found that the minimum amount of water added for GQD decoction was nine times the amount of the drug mass, and the time of decoction was 50 min; otherwise, it affected the precipitation of the active ingredients of the drug, thus affecting the efficacy (20). Its key constituent flavonoids, alkaloids, and saponins were identified through ultra-high-performance liquid chromatography and mass spectrometry (21). It has the effect of clearing the liver, diarrhea, heat, and intestines, and can be applied to diseases such as acute gastroenteritis, ulcerative colitis, gastroparesis, colon cancer, diabetes mellitus with lower limb vasculopathy, and peripheral neuropathy (22–26). Studies have shown its capacity to modulate gut flora via various molecular mechanisms, along with its beneficial effects in insulin resistance, glucose and lipid regulation, anti-inflammatory actions, and antioxidative properties (15, 16, 27, 28). A 2017 meta-analysis comparing the efficacy and safety of metformin versus metformin combined with GQD in lowering glycemia by enrolling five studies including 499 patients with T2DM found that metformin with GQD had a synergistic effect on glycemic control when compared to treatment with metformin alone (29). However, it included a small number of literatures with small sample size and outcome indicators. Our study aims to furnish evidence-based medical insights into its role in T2DM management by comprehensively reviewing randomized clinical trials (RCTs) employing Cochrane systematic evaluation methodologies.

## 2 Materials and methods

The reports in this paper are consistent with the Preferred Reporting Items for Systematic Reviews and Meta-analyses (PRISMA). Our protocol was registered and published on PROSPERO [CRD42023413758] with the title **“**The efficacy and safety of Gegen Qinlian decoction in the treatment of type 2 diabetes mellitus: a systematic review and meta-analysis of randomized clinical trials”.

### 2.1 Search strategy

We retrieved from PubMed, EMBASE, Cochrane Library, Web of Science, Scopus, Wan Fang Database, China Science and Technology Journal Database (VIP), China National Knowledge Infrastructure (CNKI), and China Medical Biological Literature Database (CMB) from the time of inception to 20 March 2023. In addition, we scoured ongoing studies on the World Health Organization (WHO) International Clinical Trials Registry Platform (ICTRP), ClinicalTrials, and the China Clinical Trials Registry (CHiCTR). The search terms mainly included “gegen qinlian”, “Gegen Qinlian decoction”, “Gegen Qinlian tang”, “Type 2 Diabetes Mellitus”, “Type 2 Diabetes”, and “Diabetes Mellitus, Non-Insulin Dependent”. The detailed search strategy for the search terms is described in **Supplementary Material**.

### 2.2 Inclusion and exclusion criteria

The inclusion criteria were as follows. (1) For the study design, all published RCTs of GQD or modified GQD for the treatment of patients with T2DM were included. Publication language was limited to English or Chinese. (2) The study objects were individuals with a diagnosis of T2DM in adults (18 years of age or older). (3) For the study intervention, GQD or modified GQD was used in the treatment group in any dosage form and amount. The control group was provided with placebo or the conventional treatment, including health education, dietary management, exercise intervention, blood glucose monitoring, and hypoglycemic medication. The treatment group may also use interventions from the control group, but must be consistent with the control group. (4) For the study outcomes, primary outcomes were fasting blood glucose (FBG), 2-h postprandial glucose (2hPG), and glycosylated hemoglobin (HbA1c); secondary outcomes were total cholesterol (TC), triglycerides (TG), high-density lipoprotein cholesterol (HDL-C), low-density lipoprotein cholesterol (LDL-C), and homeostasis model assessment of insulin resistance (HOMA-IR). Safety outcomes were any adverse events.

The exclusion criteria were as follows: (1) non-RCTs, conference abstracts, cohort studies, cross-sectional studies, case reports, guidelines, animal studies, and review articles; (2) other types of diabetes mellitus and patients with acute metabolic disorders, severe hepatic or renal impairment, severe cardiovascular disease, pregnancy, or lactation; (3) as to intervention, exclude studies using traditional Chinese medicine (TCM) treatments other than GQD; (4) for outcomes, those with incorrect data and incomplete measurement of results; and (5) repeated articles.

### 2.3 Study selection and data extraction

A database was created using EndNote X20 to manage and filter database records. Data were extracted by two authors (Yi-Mei Tan and Shuang-Hua Liu), and any inconsistencies were resolved after a debate with a third investigator (Qi-Zhi Tang). Data extraction items included first author, year of publication, study design, diagnostic criteria, sample size, sex, average age, duration of illness, duration of treatment, interventions, outcomes, comorbidities, and adverse events.

### 2.4 Quality assessment

Two authors (Yi-Mei Tan and Shuang-Hua Liu) independently assessed the risk of bias using the Risk of Bias Assessment Tool from the Cochrane Handbook. Disagreement was resolved through discussion with another reviewer (Qi-Zhi Tang). The risk of bias was assessed through seven dimensions: (1) random sequence generation, (2) allocation concealment, (3) blinding of participants and personnel, (4) blinding of outcome assessments, (5) incomplete outcome data, (6) selective reporting, and (7) other biases.

### 2.5 Statistical analysis

Meta-analysis was performed using RevMan 5. 3 and Stata 14.0. Relative risk ratios (RR) were used for dichotomous variables. For continuous variables, mean difference (MD) was used when the units of the outcome indicator were the same; otherwise, standardized mean difference (SMD) was used, and 95% confidence intervals (CI) were given. The chi^2^ test and I^2^ test were used to test for heterogeneity among studies. If p ≤ 0.05 and I^2^≥ 50%, this indicates statistically significant heterogeneity between studies; thus, a random effects model was used. We planned to explore the sources of heterogeneity and judge the stability by performing subgroup analyses and sensitivity analyses. We performed meta-regressions on the outcome metrics including more than 10 studies (FBG, 2hPG, and HbA1c) in terms of sample size, year of publication, and average age. In addition, we performed funnel plot and Egger’s linear regression tests for publication bias for FBG, 2hPG, and HbA1c, and p<0.05 was considered statistically significant, indicating possible publication bias. We conducted subgroup analyses based on the following predefined subgroup hypotheses: (1) average age (≤60 years or >60 years), (2) duration of T2DM (≤5 years or >5 years), and (3) duration of treatment (≤2 months or >2 months).

## 3 Results

### 3.1 Search results

**Figure 1:**
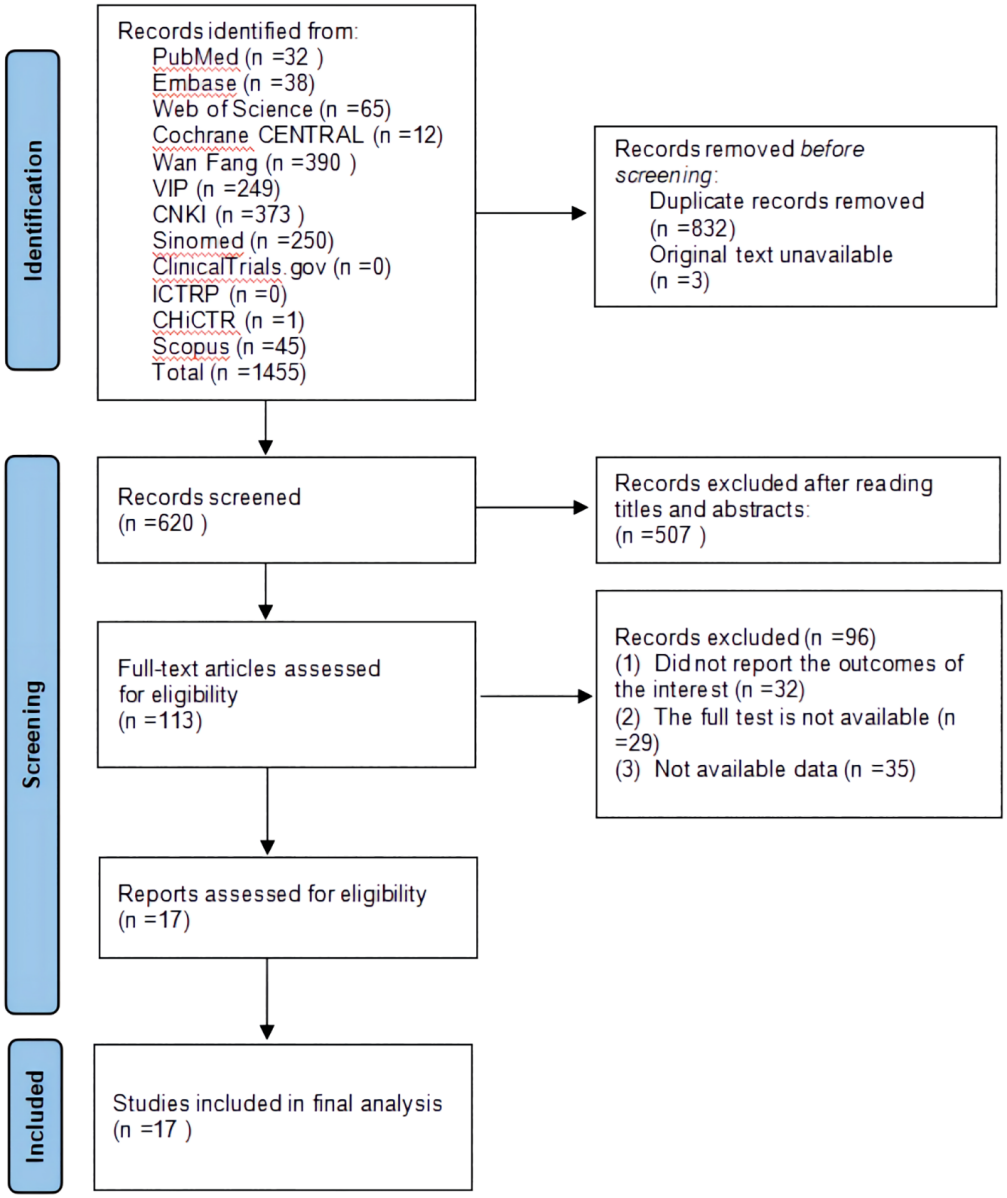
Flowchart of study identification and selection.

A total of 1,455 studies were retrieved through a database search. Three original texts were not available because they were missing from the database export. After removing 832 duplicates, 507 studies were excluded after checking the titles and abstracts of 620 citations. The full text of the remaining 113 studies was read, and 96 studies were excluded based on inclusion and exclusion criteria. Ultimately, 17 eligible studies were included in the quantitative analysis. Details of the literature screening procedure are shown in **Figure 1**.

### 3.2 Characteristics of the included studies

A total of 17 studies including 1,476 patients with T2DM were included, 830 men and 646 women (30–46). All studies were conducted in China and spanned from 2012 to 2022. The average age of the participants ranged from 36.4 to 71.9 years old, the duration of the disease ranged from 0.26 to 12.5 years, and the treatment period varied from 3 weeks to 3 months. There were nine studies with treatment groups using the original GQD; the others used modified GQD. The composition of GQD or modified GQD is shown in **Supplementary Material**. There were 13 studies where the treatment group was treated with GQD or modified GQD combined with conventional treatment. While there were three studies that the treatment group was treated with GQD or modified GQD. Only one study adopted a placebo-controlled clinical trial design approach. GQD or modified GQD is prescribed as one dose once or twice a day. Doses of *s* range from 9 g to 60 g, *Scutellaria baicalensis* and Rhizoma Coptidis from 6 g to 22.5 g, and licorice from 4 g to 15 g. The basic characteristics of the included studies are shown in **Table 1**.

Table 1The characteristics of the included studies.

**Table:** 

Study	Chen FM (2022) [30]	Chen XH (2022) [31]	Fu YH (2016) [32]	Fan YF (2017) [33]	Gong J (2019) [34]	Jin J (2019) [35]
**Study design**	RCT	RCT	RCT	RCT	RCT	RCT
**Diagnostic criteria**	2017 CDS	2017 CDS	1999 WHO	1999 WHO	NR	1999 WHO
**Sample size** **(randomized/analyzed) (E/C)**	76/76; 38/38	80/80; 40/40	90/81; 41/40	70/70; 35/35	60/60; 30/30	110//110; 55/55
**Gender (M/F) (E/C)**	20/18; 21/17	24/16; 23/17	20/21; 23/17	21/14; 19/16	17/13; 15/15	29/26; 26/29
**Average age (years) (E/C)**	45.46 ± 3.11; 45.52 ± 3.25	71.23 ± 1.94; 71.02 ± 1.83	49.5; 59.5	36.4 ± 7.1; 38.0 ± 6.5	55.78 ± 4.27; 55.61 ± 4.06	55.41 ± 9.48; 53.84 ± 10.54
**Course of disease (years) (E/C)**	2.98 ± 0.12; 2.99 ± 0.13	4.57 ± 1.71; 4.83 ± 1.65	7.5; 7.5	0.26 ± 0.14; 0.28 ± 0.13	5.22 ± 1.60; 5.19 ± 1.66	NR
**Treatment duration**	8 weeks	12 weeks	8 weeks	8 weeks	8 weeks	8 weeks
**Co-intervention**	Diet and exercise	Diet and exercise	Diet and exercise	Diet and exercise	NR	Diet
**Treatment group** **interventions**	GQD 1 dose/per day, bid + CG	Modified GQD 1 dose/per day, bid + CG	Modified GQD, qd + CG	GQD 1 dose/per day, bid	GQD 1 dose/per day, bid + CG	GQD 1 dose/per day, bid
**Control group interventions**	No drug	Metformin, 0.25g, tid	Metformin, 0.5g, tid	Metformin, 0.85g, bid	Saxagliptin, 5mg, qd	Metformin, 0.5g, tid
**Outcome index**	①②③④⑦	①②③	①②③④⑤⑥⑦	①②③④⑦⑧	①②③④⑤⑦	①②③④⑤⑦
**Baseline difference**	NSD	NSD	NSD	NSD	NSD	NSD
**Country**	China	China	China	China	China	China
**Funding**	NR	NR	NR	National Youth Natural Science Funding Project (81603585)	NR	Zhejiang Provincial Medical and Health Science and Technology Program Project (2017ZD007)
**Jadad score**	3	3	6	2	2	2

**Table:** 

Study	Li L (2020) [36]		Wang L (2021) [37]	Wang QY (2021) [38]		Wang Y (2020) [39]	Wu L (2021) [40]		Xiong QJ (2019) [41]
**Study design**	RCT		RCT	RCT		RCT	RCT		RCT
**Diagnostic criteria**	2017 CDS		2011 CDS	2017 CDS		NR	2017 CDS		2013 CDS
**Sample size** **(randomized/analyzed) (E/C)**	88/88; 44/44		100/100; 50/50	80/80; 40/40		80/80; 40/40	60/56; 27/29		100/100; 50/50
**Gender (M/F) (E/C)**	23/21; 22/22		30/20; 29/21	23/17; 22/18		22/18; 23/17	13/14; 15/14		29/21; 30/20
**Average age (years) (E/C)**	52.13 ± 3.26; 52.07 ± 3.72		49.36 ± 4.64; 48.97 ± 4.52	55.75 ± 3.56; 56.46 ± 3.35		54.4 ± 3.6; 53.6 ± 3.2	64.74 ± 10.05; 66.14 ± 9.16		53.65 ± 7.65; 53.50 ± 7.80
**Course of disease (years) (E/C)**	1.51 ± 0.49; 1.29 ± 0.33		0.40 ± 0.03; 0.40 ± 0.02	2.23 ± 1.16; 2.14 ± 1.01		4.9 ± 1.2; 4.8 ± 1.3	2; 2		4.85 ± 1.05; 4.75 ± 1.10
**Treatment duration**	3 months		3 months	3 months		3 weeks	8 weeks		8 weeks
**Co-intervention**	Diet and exercise		Diet and exercise	Diet and exercise		NR	Diet and exercise		Diet and exercise
**Treatment group interventions**	Modified GQD 1 dose/per day, bid		Modified GQD 1 dose/per day, bid + CG	GQD 1 dose/per day, bid + CG		Modified GQD 1 dose/per day, bid + CG	Modified GQD 1 dose/per day, bid + CG		Modified GQD 1 dose/per day, bid + CG
**Control group** **interventions**	Metformin, 0.5g, tid		Metformin, 0.25-0.5g, bid/tid	Sitagliptin Phosphate Tablets, 100mg, qd		Metformin, 0.5g, tid	Metformin, 0.5g, bid		Metformin, 0.25g, tid
**Outcome index**	①③④⑤⑥⑦⑧		①②③④⑤⑥⑦⑧	①②③		①②③	①②③⑧		①②③④⑤⑦
**Baseline difference**	NSD		NSD	NSD		NSD	NSD		NSD
**Country**	China		China	China		China	China		China
**Funding**	NR		NR	NR		NR	NR		NR
**Jadad score**	3		3	3		3	5		3

**Table:** 

Study		Zhang J (2018) [42]			Zhang LN (2019) [43]			Zhong XF (2021) [44]			Zhou A (2012) [45]	Zhou XY (2020) [46]
**Study design**		RCT			RCT			RCT			RCT	RCT
**Diagnostic criteria**		NR			2013 CDS			2017 CDS			1999 WHO	2017 CDS
**Sample size** **(randomized/analyzed) (E/C)**		95/95; 48/47			172/172; 86/86			40/40; 20/20			98/98; 50/48	90/90; 45/45
**Gender (M/F) (E/C)**		26/22; 25/22			51/35; 53/33			12/8; 13/7			33/17; 30/18	26/19; 22/23
**Average age (years) (E/C)**		51.3 ± 6.8; 51.2 ± 7.3			48.28 ± 10.92; 48.74 ± 11.08			71.90 ± 1.05; 71.87 ± 1.03			54.16 ± 8.18; 50.73 ± 9.40	44.2 ± 8.2; 46.1 ± 7.8
**Course of disease (years) (E/C)**		5.4 ± 2.3; 5.6 ± 2.1			NR			5.13 ± 0.35; 5.12 ± 0.34			2.14 ± 2.63; 1.44 ± 1.77	12.5 ± 6.4; 11.4 ± 5.4
**Treatment duration**		8 weeks			8 weeks			3 months			3 months	12 weeks
**Co-intervention**		NR			Diet and exercise			Diet and exercise			Diet and exercise	Diet and exercise
**Treatment group interventions**		GQD 1 dose/per day + CG			GQD 1 dose/per day, bid + CG			Modified GQD 1 dose/per day, bid + CG			GQD 1 dose/per day, bid	GQD 1 dose/per day, bid + CG
**Control group interventions**		Metformin, 0.5g, qd			Metformin, 0.25-0.5g, bid/tid			Intensive insulin therapy, 1 month; Metformin, 0.5g, bid, 2 months			placebo 1 dose/per day, bid	Liraglutide 0.6 mg in week 1, and 1.2 mg from week 2.
**Outcome index**		①②③④⑤⑥⑦			①②③⑧			①②③			①②③④⑤⑥⑦	①②③④⑤⑦⑧
**Baseline difference**		NSD			NSD			NSD			NSD	NSD
**Country**		China			China			China			China	China
**Funding**		NR			NR			NR			NR	Natural Science Foundation of Hubei Province (2018CFB546)
**Jadad score**		3			3			3			6	4

E Experimental group; C Control group; M male; F female; RCT, randomized controlled trial; ADA, american diabetes association; WHO, world health organization; CDS, chinese diabetes society; NR, not reported; GQD, gegen qinlian decoction; CG, control group interventions; NSD, no significant difference; TCM, traditional Chinese medicine; Outcome index: ①FBG; ②2hPG; ③HbA1c; ④TC; ⑤TG; ⑥HDL-C; ⑦LDL-C; ⑧HOMA-IR.

### 3.3 Risk of bias assessment

**Figure 2:**
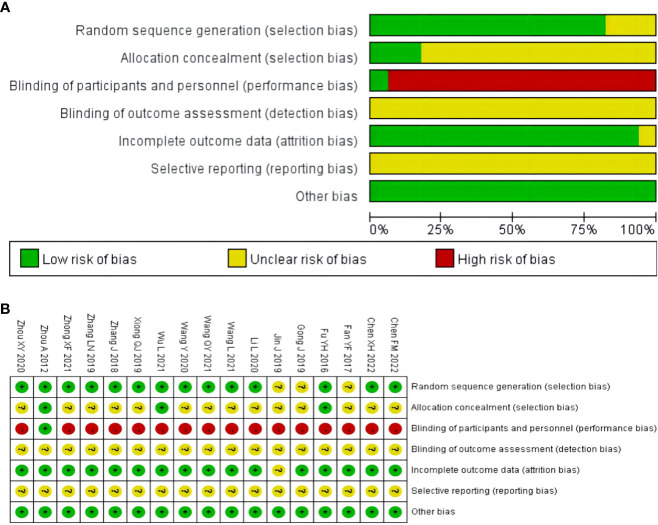
Risk of bias assessment for included studies: **(A)** risk of bias graph; **(B)** risk of bias summary.

Three studies did not report the specific methodology used in the generation of the randomized sequences (33–35). Randomization method of allocation concealment was described in three studies (32, 40, 45). One study used a stratified randomized, double-blind, placebo-controlled, multicenter clinical trial design methodology (45). One study did not report the number of patients at the end of the study (35). None of the studies described the blinding of outcome assessment, selective reporting. Overall, the methodological quality of the included literature was suboptimal. The results of the risk of bias assessment of the included studies are shown in **Figure 2**.

### 3.4 Outcomes

#### 3.4.1 GQD combined with conventional treatment vs. conventional treatment

##### 3.4.1.1 FBG

**Figure 3:**
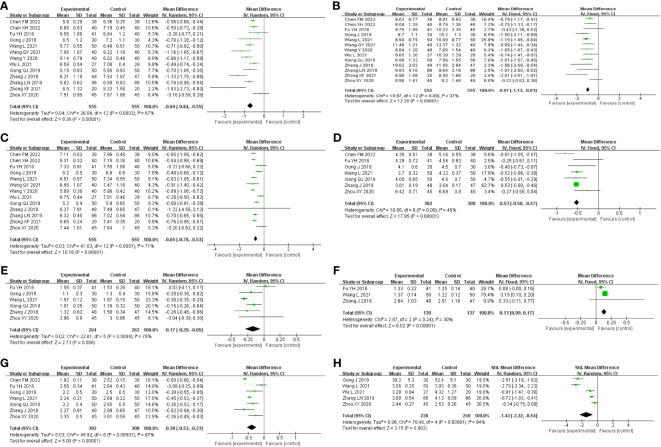
Forest plot of the GQD combined with conventional treatment vs. conventional treatment **(A)** FBG; **(B)** 2hPG; **(C)** HbA1c; **(D)** TC; **(E)** TG; **(F)** HLD-L; **(G)** LDL-C; and **(H)** HOMA-IR.

**Figure 4:**
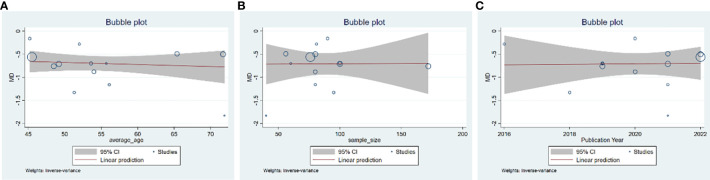
Meta-regression of the FBG for GQD combined with conventional treatment vs. conventional treatment: **(A)** average age; **(B)** sample size; and **(C)** publication year.

**Figure 5:**
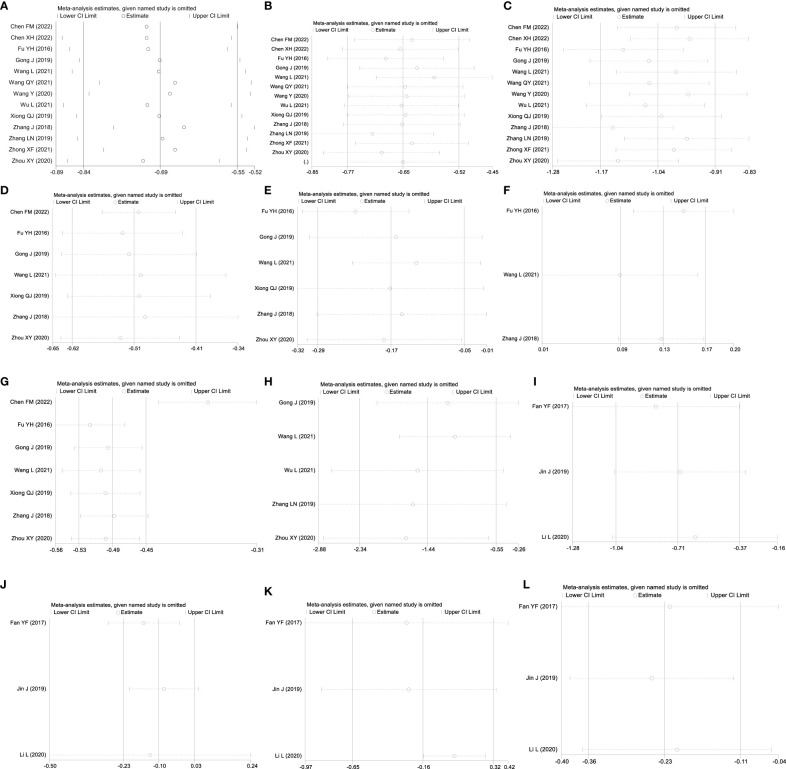
Sensitivity analysis: GQD combined with conventional treatment vs. conventional treatment: **(A)** FBG; **(B)** 2hPG; **(C)** HbA1c; **(D)** TC; **(E)** TG; **(F)** HLD-L; **(G)** LDL-C; and **(H)** HOMA-IR. GQD vs. conventional treatment: **(I)** FBG; **(J)** HbA1c; **(K)** TC; and **(L)** LDL-C.

A total of 13 studies were included, comprising 1,110 patients with T2DM. According to the heterogeneity test (p<0.01, I^2^ = 67%), a random effects model was selected for statistical analysis. It showed that GQD combined with conventional treatment resulted in lower FBG compared to conventional treatment (MD=−0.69 mmol/L, 95% CI −0.84 to −0.55, p<0.01) (**Figure 3A**). We conducted meta-regressions; the results showed that there were no significant differences in FBG by average age (p=0.597), sample size (p=0.971), or year of publication (p=0.934) (**Figure 4**). The results of subgroup analyses showed that heterogeneity within each subgroup was not completely reduced. BMI and comorbidities may be a source of heterogeneity, and more higher-quality studies need to be conducted to prove it. We also conducted sensitivity analyses, and the results were shown to be robust (**Figure 5A**).

##### 3.4.1.2 2hPG

All 13 studies were included. According to the heterogeneity test (p=0.09, I^2^ = 37%), a fixed-effects model was selected. The results showed that GQD combined with conventional treatment resulted in a reduction in 2hPG compared to conventional treatment group (MD=−0.97 mmol/L, 95% CI −1.13 to −0.81, p<0.01) (**Figure 3B**). GQD may reduce 2hPG in patients with different treatment durations, disease duration, and ages. We also performed sensitivity analyses, and the results were robust (**Figure 5B**).

##### 3.4.1.3 HbA1c

**Figure 6:**
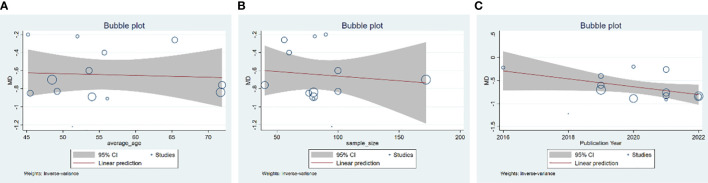
Meta-regression of the HbA1c for GQD combined with conventional treatment vs. conventional treatment: **(A)** average age; **(B)** sample size; and **(C)** publication year.

A total of 13 studies were included. According to the heterogeneity test (p<0.01, I^2^ = 71%), a random effects model was selected. The results showed that GQD combined with conventional treatment reduced HbA1c compared with conventional treatment (MD=−0.65%, 95% CI −0.78 to −0.53, p<0.01) (**Figure 3C**). Meta-regression results showed no significant differences in HbA1c by average age (p=0.815), sample size (p=0.651), or year of publication (p=0.072) (**Figure 6**). The heterogeneity within each subgroup was still high. We assumed that levels of pancreatic islet function may also be a source of heterogeneity. More high-quality studies are needed to further substantiate this. We performed sensitivity analyses, and the results were shown to be robust (**Figure 5C**).

##### 3.4.1.4 TC

A total of seven studies containing 602 patients were included. A fixed-effects model was selected according to the heterogeneity test (p=0.09, I^2^ = 45%). The results showed that GQD combined with conventional treatment was superior to conventional treatment in lowing TC (MD=−0.51 mmol/L, 95% CI −0.62 to −0.41, p < 0.01) (**Figure 3D**). Subgroup analyses showed that GQD reduced TC in different disease duration and treatment times. We also performed sensitivity analyses, by excluding the study (25); I^2^ was reduced from 45% to 1%, but the pooled results were unchanged. The results were shown to be robust (**Figure 5D**).

##### 3.4.1.5 TG

A total of six studies containing 526 patients were included. A random effects model was selected based on the heterogeneity test (p<0.01, I^2^ = 78%). The results showed that GQD combined with conventional treatment was superior in the reduction in TG (MD=−0.17 mmol/L, 95% CI −0.29 to −0.05, p<0.01) (**Figure 3E**). Subgroup analyses showed no statistically significant difference in TG lowering within the subgroup disease duration >5 years and treatment duration >2 months, suggesting that these factors may be a source of heterogeneity. Sensitivity analyses showed similar pooled effect sizes, and the results were robust (**Figure 5E**).

##### 3.4.1.6 HDL-C

A total of three studies with 276 patients were included. A fixed-effects model was selected based on the heterogeneity test (p=0.24, I^2^ = 30%). The results showed that GQD combined with conventional treatment significantly improved HDL-C (MD=0.13 mmol/L, 95% CI 0.09–0.17, p<0.01) (**Figure 3F**). Sensitivity analysis shows the results to be robust (**Figure 5F**).

##### 3.4.1.7 LDL-C

A total of seven studies including 602 patients were included. A random-effects model was selected according to the heterogeneity test (p<0.01, I^2^ = 87%). The results showed that the GQD combined with conventional treatment was superior to the reduction in LDL-C (MD=−0.38 mmol/L, 95% CI −0.53 to −0.23, p<0.01) (**Figure 3G**). Subgroup analysis showed that GQD combined with conventional treatment reduced LDL-C in different disease duration and treatment times, but heterogeneity remained high. Sensitivity analyses showed the results to be robust (**Figure 5G**).

##### 3.4.1.8 HOMA-IR

A total of five studies including 478 patients were included. A random effects model was selected based on the heterogeneity test (p<0.01, I^2^ = 94%). The results showed that GQD combined with conventional treatment had advantages in improving HOMA-IR (SMD=−1.43, 95% CI −2.32 to −0.54, p<0.01) (**Figure 3H**). Within the subgroups with a disease duration >5 years and a treatment duration >2 months, the effect size was not statistically significant, but heterogeneity was still large. We supposed that individual differences and measurement bias may be associated with heterogeneity. We performed sensitivity analyses, which turned out to be robust (**Figure 5H**).

#### 3.4.2 GQD vs. conventional treatment

##### 3.4.2.1 FBG

**Figure 7:**
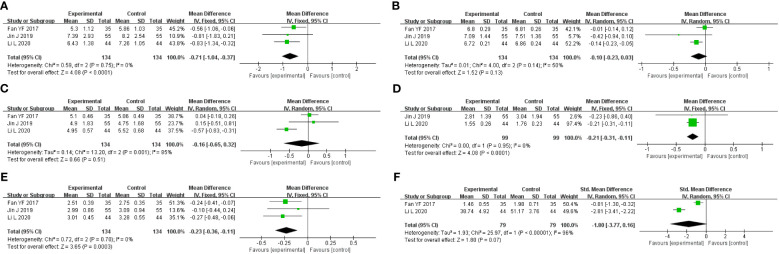
Forest plot of GQD vs. conventional treatment: **(A)** FBG; **(B)** HbA1c; **(C)** TC; **(D)** TG; **(E)** LDL-C; and **(F)** HOMA-IR.

A total of three studies were included, containing 268 patients. According to the heterogeneity test (p=0.75, I^2^ = 0%), a fixed-effects model was selected for statistical analysis. The results showed that GQD led to a reduction in FBG compared to conventional treatment (MD=−0.71 mmol/L, 95% CI −1.34 to −0.32, p < 0.01) (**Figure 7A**). Within the disease duration subgroup, a study (30) was not statistically significant, probably because of its lack of data on disease duration, which did not clear its effect on FBG. We conducted sensitivity analyses, and the results showed that the study is robust (**Figure 5I**).

##### 3.4.2.2 2hPG

One study included 110 patients, which showed that there was no significant difference between GQD and conventional treatment in lowering 2hPG (MD=−1.17 mmol/L, 95% CI −2.81 to 0.4, p=0.16).

##### 3.4.2.3 HbA1c

A total of three studies were included. According to the heterogeneity test (p=0.14, I^2^ = 50%), a random effects model was selected. Results showed no significant difference in the reduction in HbA1c in two groups (MD=−0.10%, 95%CI −0.23 to 0.03, p=0.13) (**Figure 7B**). According to subgroup analysis, treatment time may be a source of heterogeneity. We performed sensitivity analyses (**Figure 5J**), by excluding the literature (28); I^2^ was decreasing from 50% to 6%, with a reversal of the pooled result (MD=−0.16, 95% CI −0.29 to −0.03, p=0.02), suggesting that the result is not robust. This study (28) has a larger weight in the pooled results due to its narrower confidence intervals and smaller standard deviation. Therefore, perhaps GQD is superior to conventional treatment in lowering HbA1c; more studies are needed to confirm this.

##### 3.4.2.4 TC

A total of three studies were included. A random-effects model was selected for statistical analysis according to the heterogeneity test (p=0.001, I^2^ = 85%). There was no significant difference in TC reduction in two groups (MD=−0.16 mmol/L, 95% CI −0.65 to 0.32, p=0.51) (**Figure 7C**). Within the subgroups of different treatment durations, a study (31) showed that GQD was superior in lowering TC (p<0.01), and heterogeneity was significantly reduced between groups. Treatment duration may be a source of heterogeneity. We performed sensitivity analyses, by excluding the study (31); I^2^ was reduced from 85% to 0%, and the pooled results were unchanged. The results were shown to be robust (**Figure 5K**).

##### 3.4.2.5 TG

A total of two studies with 198 patients were included. A fixed-effects model was selected for statistical analysis according to the heterogeneity test (p=0.95, I^2^ = 0%). The results showed that GQD was more advantageous than conventional treatment in lowering TG (MD=−0.21 mmol/L, 95% CI −0.31 to −0.11, p<0.01) (**Figure 7D**). Switching to a random effect model did not change the significance of the result, suggesting that the result was robust.

##### 3.4.2.6 HDL-C

A study including 88 patients showed that GQD was superior in improving HDL-C (MD=0.30 mmol/L, 95% CI 0.14–0.46, p<0.01).

##### 3.4.2.7 LDL-C

A total of three studies were included. A fixed-effects model was selected according to the heterogeneity test (p=0.70, I^2^ = 0%). The results showed that GQD had a stronger effect on lowering LDL-C compared with conventional treatment (MD = −0.23 mmol/L, 95% CI −0.36 to −0.11, p < 0.01) (**Figure 7E**). The study (30) showed no difference in reducing LDL-C between two groups. However, more high-quality studies are needed, limited by the number of studies and the lack of data. A sensitivity analysis was conducted, and the results showed that the study was robust (**Figure 5L**).

##### 3.4.2.8 HOMA-IR

A total of two studies containing 158 patients were included. A random-effects model was selected according to the heterogeneity test (p<0.01, I^2^ = 96%). The results showed that there was no significant difference in HOMA-IR between two groups (MD=−1.80, 95% CI −3.77 to 0.16, p=0.07) (**Figure 7F**). The results are statistically different after switching to a fixed effects model, suggesting that the results are not robust. Due to the small number of studies and the wide variation in results, we are not able to determine the efficacy of GQD in HOMA-IR.

#### 3.4.3 GQD vs. placebo

Only one study including 98 patients adopted a placebo-controlled clinical trial design approach. GQD reduced FBG and HbA1c compared to placebo, but there was no statistically significant difference in 2hPG. There was no significant difference between two groups when comparing post-treatment and baseline lipid levels.

### 3.5 Adverse events

**Figure 8:**
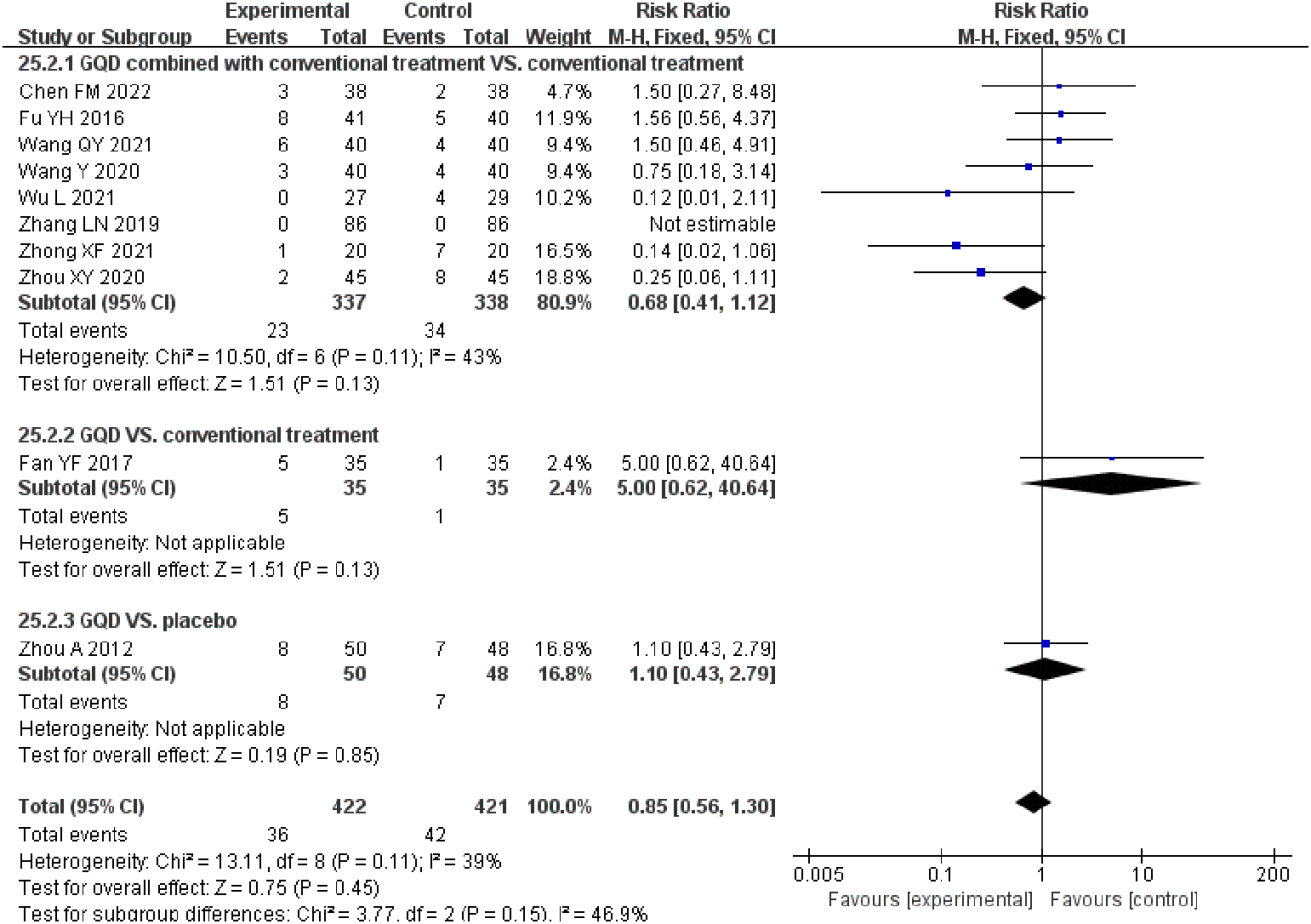
Forest plot of the adverse events.

Adverse events were reported in 10 of the 17 included studies. The results of two studies showed that adverse events were significantly lower in the combination group than in the conventional treatment group (44, 46). The results of other studies showed that the incidence of adverse events with GQD alone or in combination with conventional treatment was not significantly different from that of the conventional treatment group or the placebo group. A summary table of adverse events is available in **Supplementary Material**. The results of the meta-analysis of adverse events suggest that GQD is relatively safe (**Figure 8**).

### 3.6 Publication bias

**Figure 9:**
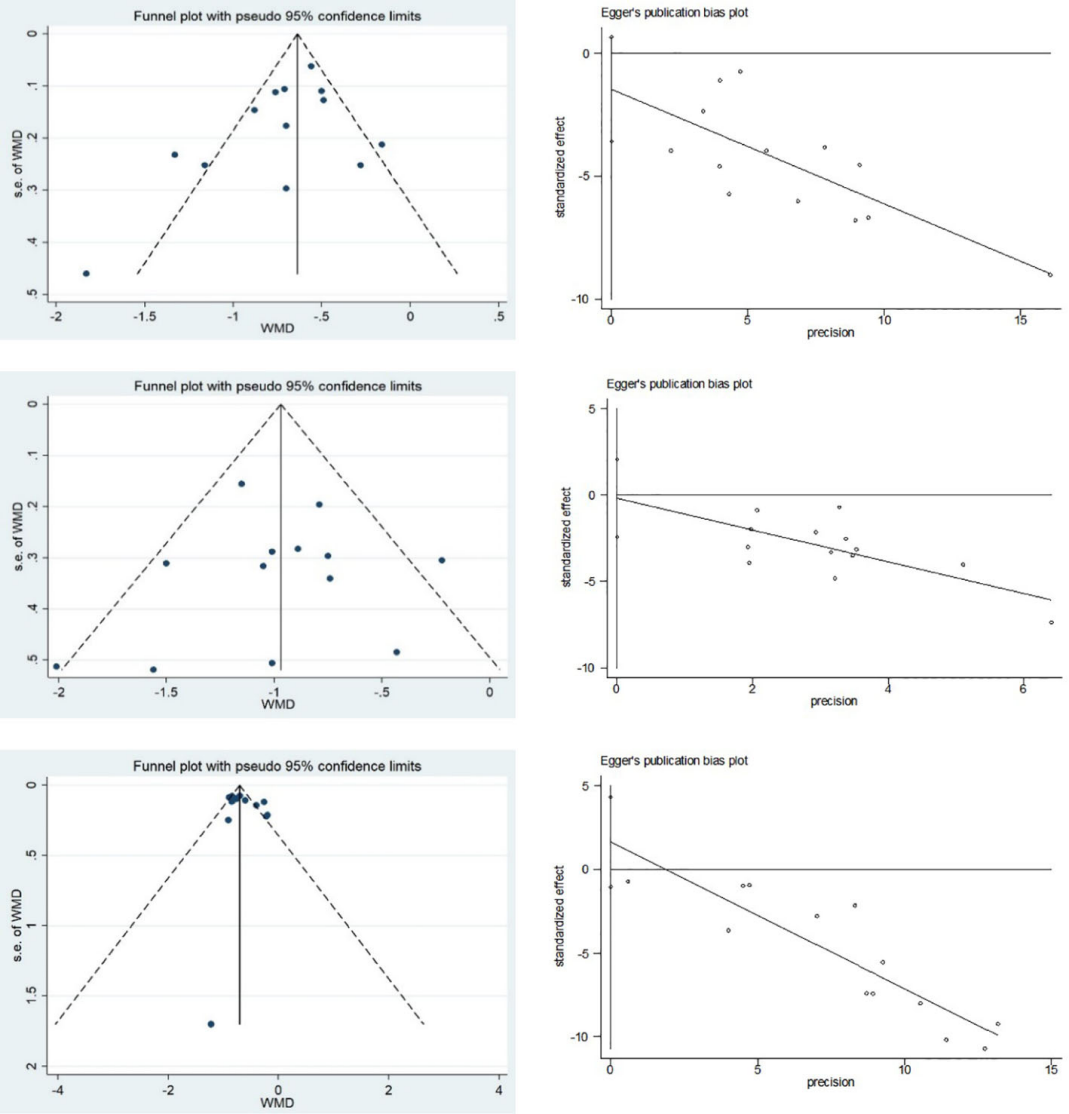
Publication bias of FBG, 2hPG and HbA1c: **(A)** Funnel plot of FBG; **(B)** Egger’s test of FBG; **(C)** Funnel plot of 2hPG; **(D)** Egger’s test of 2hPG; **(E)** Funnel plot of HbA1c; and **(F)** Egger’s test of HbA1c.

Funnel plots and Egger’s test were used to assess publication bias for FBG, 2hPG, and HbA1c (**Figure 9**). Funnel plots for HbA1c showed all but one study clustered on the top of the funnel plots, and two studies deviating from the pooled effect sizes, suggesting that there may have been heterogeneity among the studies. The Egger’s test showed no statistical difference (p=0.209), indicating that there was no significant publication bias in the studies of HbA1c. The funnel plots of FBG and 2hPG showed roughly symmetrical distributions, consistent with the results of Egger’s test (p=0.153 and 0.851, respectively), suggesting that there was no significant publication bias in the studies of FBG and 2hPG.

## 4 Discussion

### 4.1 The main results of this study

T2DM is a group of metabolic diseases characterized by chronic hyperglycemia caused by multiple etiologies, which is due to defective insulin secretion and/or utilization (47, 48). Lots of studies have found that GQD has the efficacy of regulating glycolipid metabolism and improving clinical symptoms (47, 49).

In this study, we included 17 studies and analyzed the efficacy and safety of GQD in the treatment of T2DM. Our main finding was that GQD combined with conventional treatment could reduce FBG, 2hPG, and HbA1c. GQD alone has an adjunctive hypoglycemic effect, but the quantity and quality of the included literature is small, and more high-quality studies are needed to confirm this. We suppose GQD combined with conventional treatment can be a useful complementary treatment for T2DM. There was a large heterogeneity in the results of FBG and HbA1c in the GQD combined with conventional treatment. We did not find sources of heterogeneity through meta-regression and subgroup analysis. BMI, comorbidities, and islet function are likely sources of heterogeneity. In addition, methodological shortcomings of the included studies, such as lack of blinding and allocation concealment, may have contributed to heterogeneity.

In terms of lipid metabolism, combination treatment was superior to conventional treatment in reducing TC, TG, and LDL-C and improving HDL-C. This suggests that patients with T2DM combined with abnormal lipid metabolism can benefit not only in terms of lowering blood glucose but also improving lipid metabolism with GQD combination treatment. GQD alone had some adjunctive improvement in lipid metabolism, but due to the limited number of included studies, we do not know whether GQD alone has an improved effect on lipid metabolism.

In addition, GQD combined with conventional treatment was better than conventional treatment in reducing HOMA-IR. HOMA-IR is highly heterogeneous; subgroup analyses did not identify sources of heterogeneity. We hypothesize that the heterogeneity may be related to individual differences, or it may be measurement bias caused by differences in insulin assay methods. In addition, the quality of the studies that we included was low, possibly because of the lack of blinding and allocation concealment leading to heterogeneity. The available research suggests that GQD improves islet function and controls blood glucose levels in T2DM patients (50, 51). But due to the small number of studies, it is not clear whether GQD alone has an improving effect on HOMA-IR, and more studies are needed to demonstrate this.

Adverse events were assessed in 10 of the 17 included studies. All of the adverse reactions reported in the studies resolved on their own, and no specific treatment was given. No serious adverse events were observed. The meta-analysis results suggest that GQD is relatively safe if used correctly. We performed funnel plots and Egger’s test for FBG, 2hPG and HbA1c, and no publication bias was found, suggesting that the results have some reliability.

### 4.2 Research on possible mechanisms

GQD can improve insulin resistance by activating the expression of GPR119, promoting the secretion of intestinal GLP-1 (28), increasing the serum superoxide dismutase (SOD) content, and decreasing the level of malondialdehyde (MDA) (14), and improving the inflammatory factors, such as PS, TNF-α, and IL-6, and regulating the structure of intestinal flora (16, 25, 27, 52) Through network pharmacology and bioinformatics analysis, GQD modulated 82 diabetes-related proteins and 59 diabetes-related biological pathways. Among them, modulation of the ESR1 signaling pathway plays an important role in the mechanism of GQD treatment of T2DM (53). Zhou et al. suggested that GQD may protect pancreatic islet β-cells by increasing the activity of the IRS-2/PI3K-Akt signaling pathway (54).

## 5 Limitations of this study

First, the overall quality of the included studies was poor, with the method of randomization unclear in most of them, and detailed information on blinding, allocation concealment, selective reporting, and registration of procedures was not provided. Second, the small sample sizes of the included studies and the lack of indication of the basis for sample size estimation may lead to reduced test efficacy. Third, some studies lacked baseline characteristics, such as disease duration and BMI, and most did not report comorbidities. Finally, all studies included were conducted in China, which may have potential publication bias. Due to these limitations, the standardization and overall level of clinical research must be improved. To improve the quality of reporting of RCTs, it is recommended that clinical trials be conducted in strict accordance with the latest Comprehensive Standards of Trial Reporting (CONSORT) statement.

## 6 Directions for future research

The generally low methodological quality of the studies included in this systematic evaluation, the small sample sizes of the studies, and the lack of baseline characteristics of some of the studies reduced the level of recommendation and the strength of evidence for the systematic evaluation. Therefore, future clinical study reports should pay attention to the following points. ① Clinical studies should describe in detail the specific protocol of randomization. ② There should be concealment of the randomization protocol. ③ Detailed records should be kept on the withdrawal of cases during the study period and loss of visits, and strict procedures should be established for the treatment and reporting of adverse events. ④ Due to the special characteristics of Chinese medicine soup, placebo and simulant preparation technology is still imperfect, so it is more difficult to implement the blinding method, but for granules and capsules, the blinding method should be implemented and the impact of the blinding method on the evaluation of the results could be described. Therefore, such interventions could be used in future clinical studies. ⑤ Clinical research should be carried out beforehand to estimate the sample size and explain the basis for the improvement of the test effectiveness. In addition, we should standardize the reporting of adverse reactions to Chinese medicines by adopting the method of combining disease and evidence.

## 7 Conclusion

In summary, this meta-analysis found that GQD can be used in the treatment of type 2 diabetes mellitus, which has the efficacy of assisting in lowering glucose and regulating lipids, and improving the function of pancreatic islets. Meanwhile, GQD is relatively safe. However, this finding still needs further validation due to the limit of the number of included studies, sample size, and methodology of studies, and further high-quality, large-sample, double-blind, multicenter RCTs are needed to provide more reliable evidence for the clinical application of GQD.

## Data availability statement

The original contributions presented in the study are included in the article/**Supplementary Material**. Further inquiries can be directed to the corresponding author.

## Author contributions

QT: Conceptualization, Project administration, Supervision, Writing – review & editing. YT: Data curation, Formal analysis, Methodology, Software, Writing – original draft, Writing – review & editing. SL: Data curation, Formal analysis, Investigation, Methodology, Software, Validation, Writing – original draft, Writing – review & editing. MH: Investigation, Methodology, Software, Writing – original draft. HC: Data curation, Formal analysis, Methodology, Writing – original draft. BX: Methodology, Software, Writing – review & editing. HL: Data curation, Formal analysis, Investigation, Writing – original draft.

## References

[1] Magliano DJ Boyko EJ . committee IDFDAtes: IDF diabetes atlas. Brussels International Diabetes Federation © International Diabetes Federation (2021) 2021.

[2] Wu J Gao LX . Diabetes map of China: People's health publishing house. (2022) 2022.

[3] ElSayed NA Aleppo G Aroda VR Bannuru RR Brown FM Bruemmer D . 2. Classification and diagnosis of diabetes: Standards of care in diabetes-2023. Diabetes Care (2023) 46:S19–s40. doi: 10.2337/dc23-S002 36507649 PMC9810477

[4] He J Li Z Xia P Shi A FuChen X Zhang J . Ferroptosis and ferritinophagy in diabetes complications. Mol Metab (2022) 60:101470. doi: 10.1016/j.molmet.2022.101470 35304332 PMC8980341

[5] Pop-Busui R Januzzi JL Bruemmer D Butalia S Green JB Horton WB . Heart failure: An underappreciated complication of diabetes. A consensus report of the American diabetes association. Diabetes Care (2022) 45:1670–90. doi: 10.2337/dci22-0014 PMC9726978 35796765

[6] Zheng Y Ley SH Hu FB . Global aetiology and epidemiology of type 2 diabetes mellitus and its complications. Nat Rev Endocrinol (2018) 14:88–98. doi: 10.1038/nrendo.2017.151 29219149

[7] Singh A Kukreti R Saso L Kukreti S . Mechanistic insight into oxidative stress-triggered signaling pathways and type 2 diabetes. Molecules (2022) 27(3):950–70. doi: 10.3390/molecules27030950 PMC8840622 35164215

[8] Stanaway JD Afshin A Gakidou E Lim SS Abate D Abate KH . Global, regional, and national comparative risk assessment of 84 behavioural, environmental and occupational, and metabolic risks or clusters of risks for 195 countries and territories, 1990-2017: A systematic analysis for the Global Burden of Disease Study 2017. Lancet (2018) 392:1923–94. doi: 10.1016/S0140-6736(18)32225-6 PMC6227755 30496105

[9] American Diabetes Association . Pharmacologic approaches to glycemic treatment: Standards of medical care in diabetes-2021. Diabetes Care (2021) 44:S111–s124. doi: 10.2337/dc21-S009 33298420

[10] De Jager J Kooy A Lehert P Wulffelé MG van der Kolk J Bets D . Long term treatment with metformin in patients with type 2 diabetes and risk of vitamin B-12 deficiency: randomised placebo controlled trial. Bmj. (2010) 340:c2181. doi: 10.1136/bmj.c2181 20488910 PMC2874129

[11] Kernan WN Viscoli CM Furie KL Young LH Inzucchi SE Gorman M . Pioglitazone after ischemic stroke or transient ischemic attack. N Engl J Med (2016) 374:1321–31. doi: 10.1056/NEJMoa1506930 PMC4887756 26886418

[12] American Diabetes Association . Implications of the diabetes control and complications trial. Am Diabetes Assoc Diabetes (1993) 42:1555–8. doi: 10.2337/diab.42.11.1555 8405694

[13] Stratton IM Adler AI Neil HA Matthews DR Manley SE Cull CA . Association of glycaemia with macrovascular and microvascular complications of type 2 diabetes (UKPDS 35): prospective observational study. Bmj. (2000) 321:405–12. doi: 10.1136/bmj.321.7258.405 PMC27454 10938048

[14] Lou WJ Guo J Zhang F Jiang YH Luo Q Wang YX . Systematic review and meta-analysis of the efficacy and safety of berberine (Berberine) in the treatment of early diabetic nephropathy. Chin J Integr Nephrol (2022) 23(06):510–3.

[15] Yuan Y Hou XF Feng L Jia XB Wang YQ . Inhibition of late glycosylation end-product formation by Puerarin *in vitro* and *in vivo* . Chin herbal Med (2017) 48(07):1386–90.

[16] Wang T Jiang H Cao S Chen Q Cui M Wang Z . Barcalin and its metabolites suppresses gluconeogenesis through activation of AMPK or AKT in insulin resistant HepG-2 cells. Eur J medicinal Chem (2017) 141:92–100. doi: 10.1016/j.ejmech.2017.09.049 29028535

[17] Pan JQ Han C Liu HC Du JW Li KJ . Experimental study on hypoglycemic effects of Gegen Qinlian Decoction. Chin New Drugs J (2000) 9(3):167–70.

[18] Tong XL Zhao LH Lian FM Zhou Q Xia L Zhang JC . Clinical observations on the dose-effect relationship of gegen qin lian decoction on 54 out-patients with type 2 diabetes. J Traditional Chin Med (2011) 31(01):56–9. doi: 10.1016/S0254-6272(11)60013-7 21563509

[19] Wen J Liu QH Zhang J Peng ZP Tong XL . Effects of different dosage values and decoction methods on the quality of Gegen Qin Lian Decoction. Chin J Exp Formulas. (2011) 31(01):56–9.

[20] Zhang JC Zhang J Liu F Mu LC Guo Y Wang YS . Effects of water addition and decoction time on the amount of major components dissolved in Gegen Qin Lian Decoction. Chin J Exp Formulas (2013) 19(1):13–7. doi: 10.13422/j.cnki.syfjx.2013.01.047

[21] Wang TT An H Liang K Ji WL Xu YW Lu J . Analysis of the chemical constituents of Gegen Qinlian Decoction based on UPLC-LTQ-Orbitrap high-resolution mass spectrometry. Chin Herbal Med (2020) 51:1498–507. doi: 10.7501/j.issn.0253-2670.2020.06.017

[22] Zeng YP Wang AH Hu YG . Study on the treatment of diabetic gastroparesis with damp-heat syndrome by compound prescription. Modern J Integr Chin Western Med (2006) 2023–2024+2140.

[23] Cao Q Li TX . Evaluation of clinical efficacy of Gegen Qinlian Decoction combined with lipoic acid in the treatment of diabetic peripheral neuropathy in the elderly. Chin J Traditional Chin Med (2017) 35:2443–5. doi: 10.13193/j.issn.1673-7717.2017.09.068

[24] Zheng CX Feng JX Chen ZT Guo XH Cheng NN Dai X . Mechanism of protective effect of Gegen Qinlian Decoction on intestinal barrier in mice with ulcerative colitis. Chin J Veterinary Medicine (2023) 43:571–6. doi: 10.16303/j.cnki.1005-4545.2023.03.21

[25] Xu BL Wu D Yang NN Han XY Liu JJ Hu Y . Pathway-disease interaction network-based intervention of Gegen Qinlian Decoction and its index component combinations in ulcerative colitis-associated colon cancer in mice. Chin Herbal Med (2020) 51:4991–8.

[26] Zhang GX Du HY Yang GY Su G Tian WY Tu XH . Effects of Gegen Qinlian Decoction on the expression of PINK1/Parkin in colonic tissues of a rat model of dysbiotic diarrhea. Shizhen Guomian Guomao. (2023) 34:857–61.

[27] Zhang CH Ma GQ Dang YB Wang XY Chen YC Tu XY . Effects of Gegen Qinlian Decoction on plasma LPS, TNF-α, IL-6 and intestinal flora in KK-Ay diabetic mice. Chin Herbal Med (2017) 48:1611–6.

[28] Chen J Qian ZX Lin X Zhu MY Ge YM Chen C . Experimental study on the regulation of GLP-1 secretion by Gegen Qinlian Decoction based on GPR119 expression. Shizhen Guomian Guomian (2021) 32:329–31.

[29] Ryu JA Lixia M Cao S . Efficacy and safety of Gegen Qinlian decoction for normalizing hyperglycemia in diabetic patients: A systematic review and meta-analysis of randomized clinical trials. Complement Ther Med (2017) 33:6–13. doi: 10.1016/j.ctim.2017.05.004 28735827

[30] Chen FM . Effects of gegen qinlian decoction on body mass index and blood insulin levels in pre-diabetes mellitus. Chin Sci Technol J Database (Citation Edition) Med Health (2022) 2022(8):284–86.

[31] Zheng LM Chen XH . Discussing the efficacy of modified Gegen Qinlian Decoction reduction in the treatment of gastrointestinal damp-heat type 2 diabetes mellitus in the elderly. Chin Sci Technol J Database (full text version) Med Health (2022) 2022(1):17–20.

[32] Fu YH . Therapeutic effect of Gegen Qinlian Decoction in the treatment of type 2 diabetes mellitus with dampness-heat in the spleen. Guangzhou Univ Traditional Chin Med (2016).

[33] Liu C Fan YF Cao W Hu YX Liu KG . Study on the effect of Gegen Qinlian Decoction on insulin resistance in new-onset type 2 diabetes mellitus. Modern J Integr Chin Western Med (2017) 26:115–21. doi: 10.3969/j.issn.1008-49.2017.02.001

[34] Gong J . Clinical observation on the treatment of type 2 diabetes mellitus by Gegen Qinlian Decoction combined with saxagliptin. J Pract Chin Med (2019) 35:481–2.

[35] Jin J Bao BY . Clinical study on the treatment of type 2 diabetes mellitus combined with obesity and hyperlipidemia by Gegen Qinlian Decoction, in: Proceedings of the Academic Conference of Nutrition and Metabolism Branch of Zhejiang Provincial Medical Association. (2019) pp. 444–9.

[36] Li L . Effect of Gegen Qinlian Decoction on patients with first-onset type 2 diabetes mellitus. Guangming Traditional Chin Med (2020) 35:1183–4. doi: 10.3969/j.issn.1003-8914.2020.08.026

[37] Wang L . Clinical study on the treatment of newly diagnosed type 2 diabetes mellitus with phlegm (dampness) and heat conjugation by adding Gegen Qinlian Decoction. New Chin Medicine (2021) 53:16–20. doi: 10.1186/s13020-021-00426-1

[38] Wang QY Li N Chen Y . Analysis of the effect of Gegen Qinlian Decoction combined with selegiline in the treatment of type 2 diabetes mellitus. Front Med (2021) 11:187–8.

[39] Wang Y . Effect of Gegen Qinlian Decoction on the therapeutic effect of type 2 diabetes mellitus patients. Med Equip (2020) 33:83–4.

[40] Wu L . Clinical study on the treatment of type 2 diabetes mellitus with damp-heat intermediate obstruction by addition and subtraction of Gegen Qinlian Decoction. Chongqing Med Univ (2021).

[41] Xiong QJ . Clinical effect of Gegen Qinlian Decoction in the treatment of type 2 diabetes mellitus. Clin Med Res Practice. (2019) 4:148–9. doi: 10.19347/j.cnki.2096-1413.201926063

[42] Chen R Zhang J Ma Q . Clinical effect and mechanism of action of Gegen Qinlian Decoction on type 2 diabetes mellitus combined with lower limb vasculopathy. Shaanxi Traditional Chin Med (2018) 39:86–8. doi: 10.3969/j.issn.1000-7369.2018.01.028

[43] Wei ZC Zhang LN Yang L . Evaluation of clinical efficacy of Gegen Qinlian Decoction in the treatment of type 2 diabetes mellitus. Contemp Med (2019) 25:10–2. doi: 10.3969/j.issn.1009-4393.2019.32.004

[44] Zhong XF Lin ZH . Analysis of the efficacy of Gegen Qinlian Decoction plus reduction with insulin pump and metformin in the treatment of gastrointestinal damp-heat type 2 diabetes mellitus. Diabetes New World (2021) 24:72–75,79. doi: 10.16658/j.cnki.1672-4062.2021.22.072

[45] Zhou A . Clinical study on treatment of type 2 diabetes mellitus with damp-heat trapping the spleen in gegen qinlian decoction. Beijing Univ Chin Med (2012).

[46] Zhou XY . Clinical efficacy of Gegen Qinlian Decoction combined with liraglutide in obese type 2 diabetes mellitus with dampness-heat trapped spleen syndrome. Tianjin Traditional Chin Med (2020) 37:1363–7. doi: 10.11656/j.issn.1672-1519.2020.12.09

[47] Li X Geng-Ji JJ Quan YY Qi LM Sun Q Huang Q . Role of potential bioactive metabolites from traditional Chinese medicine for type 2 diabetes mellitus: An overview. Front Pharmacol (2022) 13:1023713. doi: 10.3389/fphar.2022.1023713 36479195 PMC9719995

[48] Weisman A Fazli GS Johns A Booth GL . Evolving trends in the epidemiology, risk factors, and prevention of type 2 diabetes: A review. Can J Cardiol (2018) 34:552–64. doi: 10.1016/j.cjca.2018.03.002 29731019

[49] Xu L Li Y Dai Y Peng J . Natural products for the treatment of type 2 diabetes mellitus: Pharmacology and mechanisms. Pharmacol Res (2018) 130:451–65. doi: 10.1016/j.phrs.2018.01.015 29395440

[50] Tian J Lian F Tong X . Safety and effectiveness of different herbal medicine dosage of Gegen Qinlian Decoction in Chinese patients with type 2 diabetes: A double-blind, two-part, randomised controlled trial. Lancet Diabetes Endocrinol (2016) 4(SPEC. ISSUE 3):S25. doi: 10.1016/S2213-8587(16)30380-1

[51] Tian J Lian F Yu X Cui Y Zhao T Cao Y . The efficacy and safety of chinese herbal decoction in type 2 diabetes: A 5-year retrospective study. Evid Based Complement Alternat Med (2016) 2016:5473015. doi: 10.1155/2016/5473015 27656237 PMC5021493

[52] Xu X Niu L Liu Y Pang M Lu W Xia C . Study on the mechanism of Gegen Qinlian Decoction for treating type II diabetes mellitus by integrating network pharmacology and pharmacological evaluation. J Ethnopharmacol (2020) 262:113–29. doi: 10.1016/j.jep.2020.113129 32730886

[53] Wang L He YH Hu XY Sun YP Wang B Li N . Exploring the potential mechanism of action of Gegen Qinlian Decoction in the treatment of metabolic syndrome based on UHPLC-Q-TOF-MS/MS and network pharmacology. New Chin Medicines Clin Pharmacol (2022) 33:484–91. doi: 10.7501/j.issn.0253-2670.2020.06.017

[54] Zhou Q Zhu XD Tong XL Wang Y Si XL Wang Y . Effects of Gegen Qinlian Decoction on IRS-2/PI3K-Akt pathway in pancreatic islet cells of rats modeled with type 2 diabetes mellitus. J Traditional Chin Med (2018) 59:973–7. doi: 10.13288/j.11-2166/r.2018.11.018

