# Supplementary material for: Efficacy and safety of Gegen Qinlian decoction in the treatment of type II diabetes mellitus: a systematic review and meta-analysis of randomized clinical trials
Source: Front Endocrinol (Lausanne). 2024 Jan 26;14:1316269. doi: 10.3389/fendo.2023.1316269 (PMC10858613; doi:10.3389/fendo.2023.1316269)
Supplement: Supplementary file 1 [file DataSheet_1.pdf]

Supplementary Material 1. Database and Search Strategies

Supplementary Material 2. Components of GQD or its modified used in the included studies

Supplementary Material 3. Meta-regression analysis of FBG and HbA1c 14

Supplementary Material 4. Subgroup analysis

Supplementary Material 5. Summary of adverse reactions

Supplementary Material 6. Egger's test of HBA1c, FBG and 2hPG

Database and Search Strategies

PubMed

The retrieval of the PubMed database contained a total of 32 records.

Search	Actions	Details	Query	Results	Time
#5	•••	>	Search: ((Diabetes Mellitus, Type 2[MeSH Terms]) OR ((((((((((((((((((((((((((((((Diabetes Mellitus, Noninsulin-Dependent[Title/Abstract]) OR (Diabetes Mellitus, Ketosis-Resistant[Title/Abstract])) OR (Diabetes Mellitus, Ketosis Resistant[Title/Abstract])) OR (Ketosis-Resistant Diabetes Mellitus[Title/Abstract])) OR (Diabetes Mellitus, Non Insulin Dependent[Title/Abstract])) OR (Diabetes Mellitus, Non-Insulin-Dependent[Title/Abstract])) OR (Non-Insulin-Dependent Diabetes Mellitus[Title/Abstract])) OR (Diabetes Mellitus, Stable[Title/Abstract])) OR (Stable Diabetes Mellitus[Title/Abstract])) OR (Diabetes Mellitus, Type II[Title/Abstract])) OR (NIDDM[Title/Abstract])) OR (Diabetes Mellitus, Noninsulin Dependent[Title/Abstract])) OR (Diabetes Mellitus, Maturity-Onset[Title/Abstract])) OR (Diabetes Mellitus, Maturity Onset[Title/Abstract])) OR (Maturity-Onset Diabetes Mellitus[Title/Abstract])) OR (Maturity Onset Diabetes Mellitus[Title/Abstract])) OR (MODY[Title/Abstract])) OR (Diabetes Mellitus, Slow-Onset[Title/Abstract])) OR (Diabetes Mellitus, Slow Onset[Title/Abstract])) OR (Slow-Onset Diabetes Mellitus[Title/Abstract])) OR (Type 2 Diabetes Mellitus[Title/Abstract])) OR (Noninsulin-Dependent Diabetes Mellitus[Title/Abstract])) OR (Noninsulin Dependent Diabetes Mellitus[Title/Abstract])) OR (Maturity-Onset Diabetes[Title/Abstract])) OR (Diabetes, Maturity-Onset[Title/Abstract])) OR (Maturity Onset Diabetes[Title/Abstract])) OR (Type 2 Diabetes[Title/Abstract])) OR (Diabetes, Type 2[Title/Abstract])) OR (Diabetes Mellitus, Adult-Onset[Title/Abstract])) OR (Adult-Onset Diabetes Mellitus[Title/Abstract])) OR (Diabetes Mellitus, Adult Onset[Title/Abstract]))) AND ((((((((gegen qinlian[Title/Abstract]) OR (GeGen QinLian[Title/Abstract])) OR (Gegen Qinlian tang[Title/Abstract])) OR (Gegen Qinlian decoction[Title/Abstract])) OR (Gegenqinlian[Title/Abstract])) OR (Gegen-Qinlian[Title/Abstract])) OR (Gegen-Qin-Lian[Title/Abstract])) OR (Ge-Gen-Qin-Lian[Title/Abstract]))	32	11:19:45
#4	•••	>	Search: (((((((gegen qinlian[Title/Abstract]) OR (GeGen QinLian[Title/Abstract])) OR (Gegen Qinlian tang[Title/Abstract])) OR (Gegen Qinlian decoction[Title/Abstract])) OR (Gegenqinlian[Title/Abstract])) OR (Gegen-Qinlian[Title/Abstract])) OR (Gegen-Qin-Lian[Title/Abstract])) OR (Ge-Gen-Qin-Lian[Title/Abstract])	162	11:17:56
#3	•••	>	Search: (Diabetes Mellitus, Type 2[MeSH Terms]) OR ((((((((((((((((((((((((((((((Diabetes Mellitus, Noninsulin-Dependent[Title/Abstract]) OR (Diabetes Mellitus, Ketosis-Resistant[Title/Abstract])) OR (Diabetes Mellitus, Ketosis Resistant[Title/Abstract])) OR (Ketosis-Resistant Diabetes Mellitus[Title/Abstract])) OR (Diabetes Mellitus, Non Insulin Dependent[Title/Abstract])) OR (Diabetes Mellitus, Non-Insulin-Dependent[Title/Abstract])) OR (Non-Insulin-Dependent Diabetes Mellitus[Title/Abstract])) OR (Diabetes Mellitus, Stable[Title/Abstract])) OR (Stable Diabetes Mellitus[Title/Abstract])) OR (Diabetes Mellitus, Type II[Title/Abstract])) OR (NIDDM[Title/Abstract])) OR (Diabetes Mellitus, Noninsulin Dependent[Title/Abstract])) OR (Diabetes Mellitus, Maturity-Onset[Title/Abstract])) OR (Diabetes Mellitus, Maturity Onset[Title/Abstract])) OR (Maturity-Onset Diabetes Mellitus[Title/Abstract])) OR (Maturity Onset Diabetes Mellitus[Title/Abstract])) OR (MODY[Title/Abstract])) OR (Diabetes Mellitus, Slow-Onset[Title/Abstract])) OR (Diabetes Mellitus, Slow Onset[Title/Abstract])) OR (Slow-Onset Diabetes Mellitus[Title/Abstract])) OR (Type 2 Diabetes Mellitus[Title/Abstract])) OR (Noninsulin-Dependent Diabetes Mellitus[Title/Abstract])) OR (Noninsulin Dependent Diabetes Mellitus[Title/Abstract])) OR (Maturity-Onset Diabetes[Title/Abstract])) OR (Diabetes, Maturity-Onset[Title/Abstract])) OR (Maturity Onset Diabetes[Title/Abstract])) OR (Type 2 Diabetes[Title/Abstract])) OR (Diabetes, Type 2[Title/Abstract])) OR (Diabetes Mellitus, Adult-Onset[Title/Abstract])) OR (Adult-Onset Diabetes Mellitus[Title/Abstract])) OR (Diabetes Mellitus, Adult Onset[Title/Abstract]))	228,400	10:48:21
#2	•••	>	Search: ((((((((((((((((((((((((((((((Diabetes Mellitus, Noninsulin-Dependent[Title/Abstract]) OR (Diabetes Mellitus, Ketosis-Resistant[Title/Abstract])) OR (Diabetes Mellitus, Ketosis Resistant[Title/Abstract])) OR (Ketosis-Resistant Diabetes Mellitus[Title/Abstract])) OR (Diabetes Mellitus, Non Insulin Dependent[Title/Abstract])) OR (Diabetes Mellitus, Non-Insulin-Dependent[Title/Abstract])) OR (Non-Insulin-Dependent Diabetes Mellitus[Title/Abstract])) OR (Diabetes Mellitus, Stable[Title/Abstract])) OR (Stable Diabetes Mellitus[Title/Abstract])) OR (Diabetes Mellitus, Type II[Title/Abstract])) OR (NIDDM[Title/Abstract])) OR (Diabetes Mellitus, Noninsulin Dependent[Title/Abstract])) OR (Diabetes Mellitus, Maturity-Onset[Title/Abstract])) OR (Diabetes Mellitus, Maturity Onset[Title/Abstract])) OR (Maturity-Onset Diabetes Mellitus[Title/Abstract])) OR (Maturity Onset Diabetes Mellitus[Title/Abstract])) OR (MODY[Title/Abstract])) OR (Diabetes Mellitus, Slow-Onset[Title/Abstract])) OR (Diabetes Mellitus, Slow Onset[Title/Abstract])) OR (Slow-Onset Diabetes Mellitus[Title/Abstract])) OR (Type 2 Diabetes Mellitus[Title/Abstract])) OR (Noninsulin-Dependent Diabetes Mellitus[Title/Abstract])) OR (Noninsulin Dependent Diabetes Mellitus[Title/Abstract])) OR (Maturity-Onset Diabetes[Title/Abstract])) OR (Diabetes, Maturity-Onset[Title/Abstract])) OR (Maturity Onset Diabetes[Title/Abstract])) OR (Type 2 Diabetes[Title/Abstract])) OR (Diabetes, Type 2[Title/Abstract])) OR (Diabetes Mellitus, Adult-Onset[Title/Abstract])) OR (Adult-Onset Diabetes Mellitus[Title/Abstract])) OR (Diabetes Mellitus, Adult Onset[Title/Abstract])	176,670	10:44:19
#1	•••	>	Search: Diabetes Mellitus, Type 2[MeSH Terms]	167,974	10:30:08

Embase

The retrieval of the Embase database contained a total of 38 records.

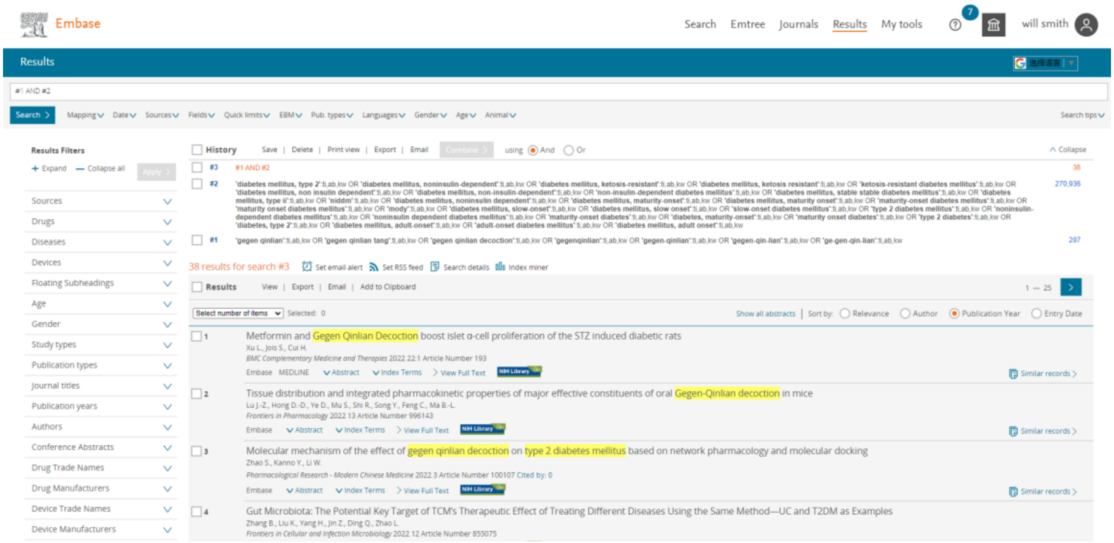

Web of Science

The retrieval of the Web of Science database contained a total of 65 records.

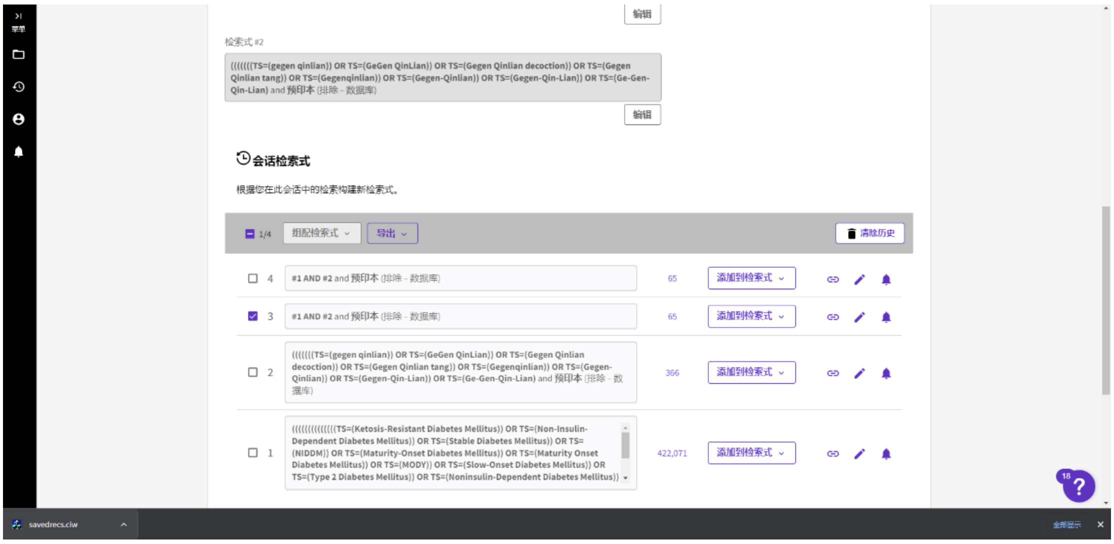

Cochrane

The retrieval of the Cochrane Library contained a total of 12 records.

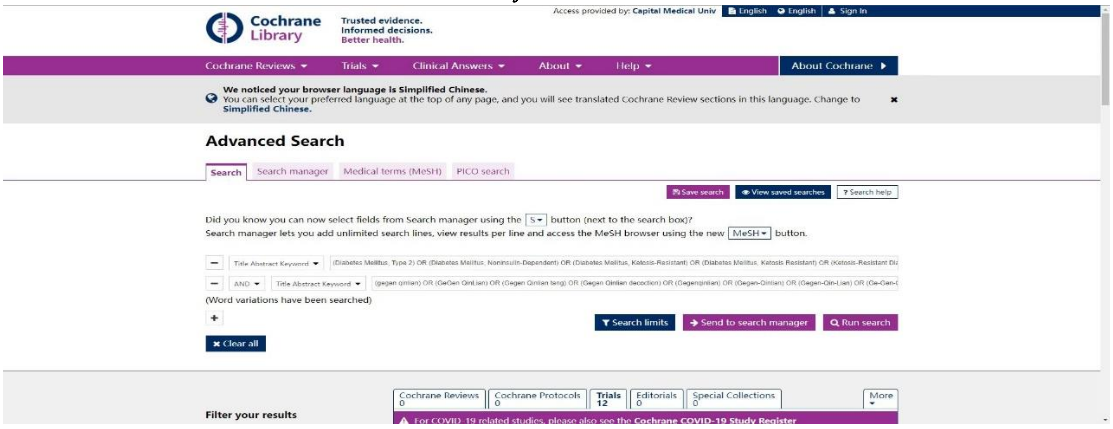

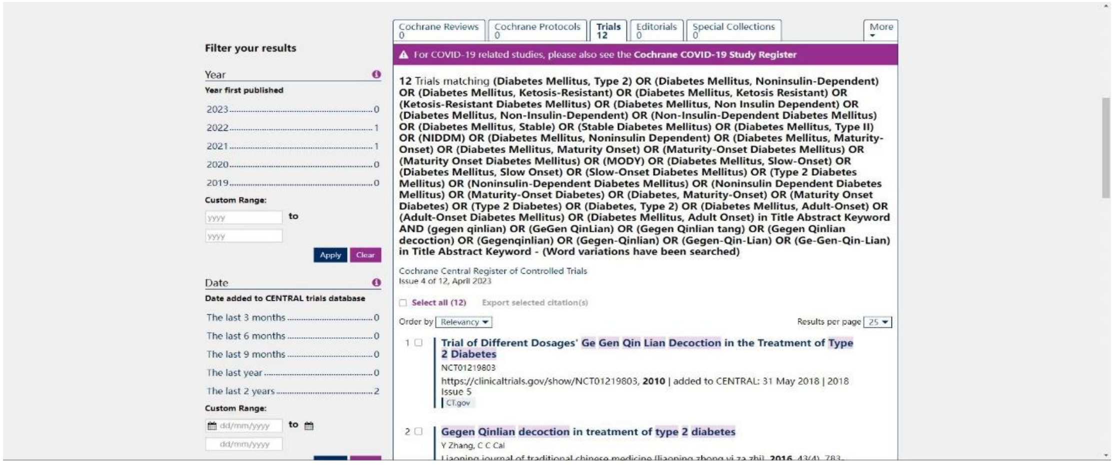

WangFang

The retrieval of the WangFang database contained a total of 390 records.

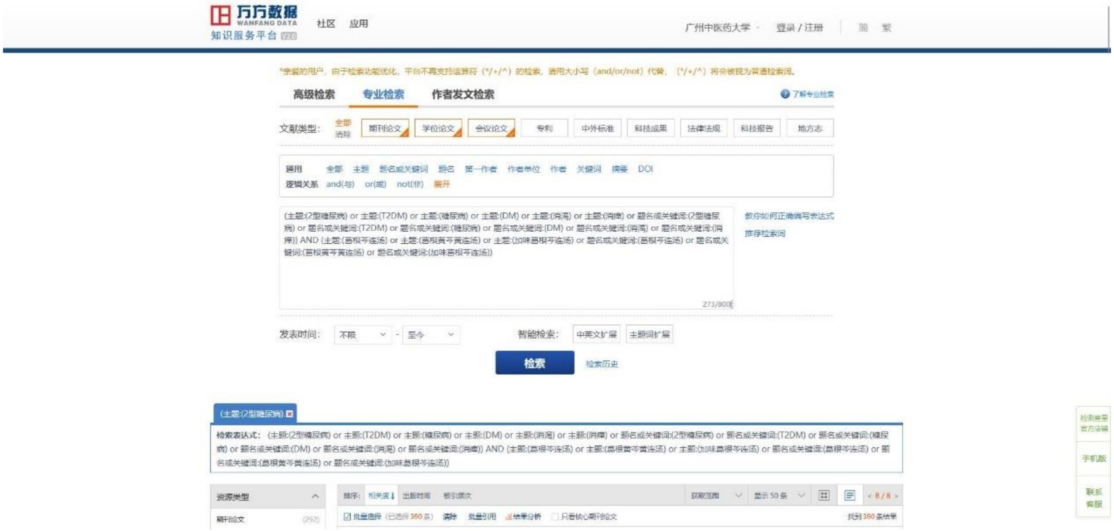

VIP

The retrieval of the VIP database contained a total of 249 records.

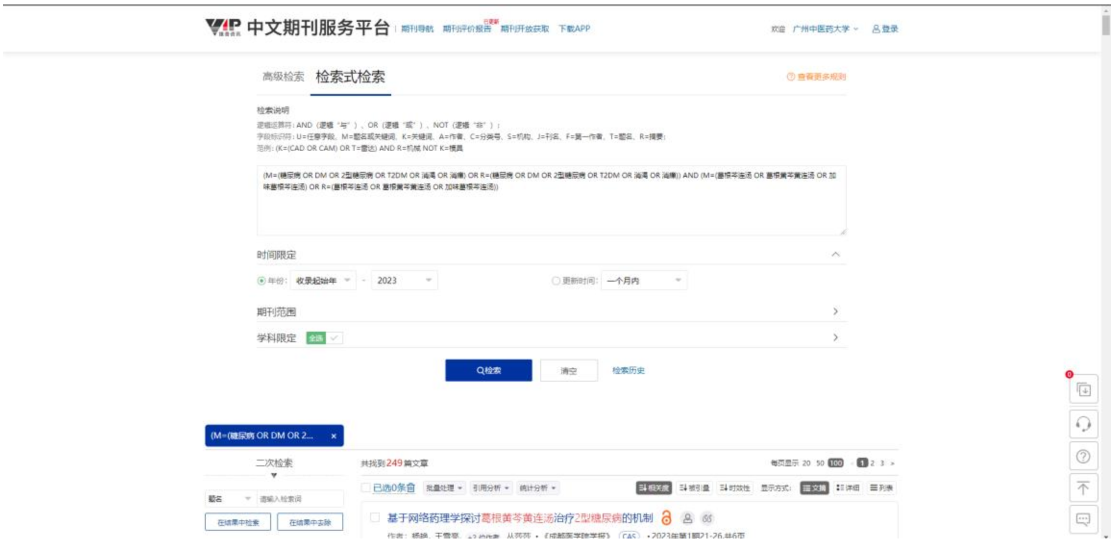

CNKI

The retrieval of the CNKI database contained a total of 373 records.

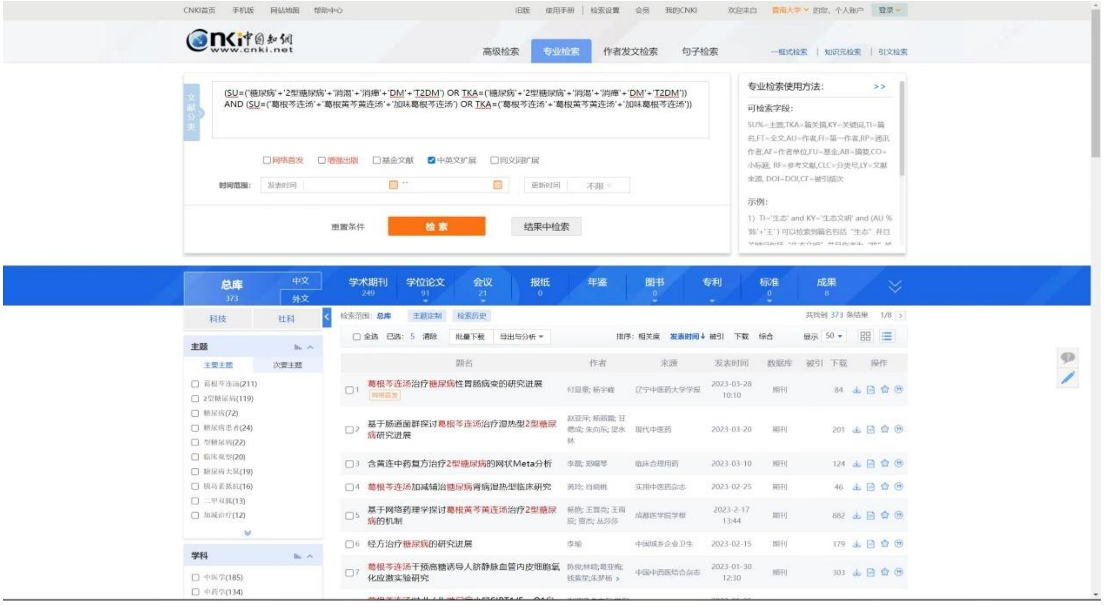

Sinomed

The retrieval of the Sinomed database contained a total of 250 records.

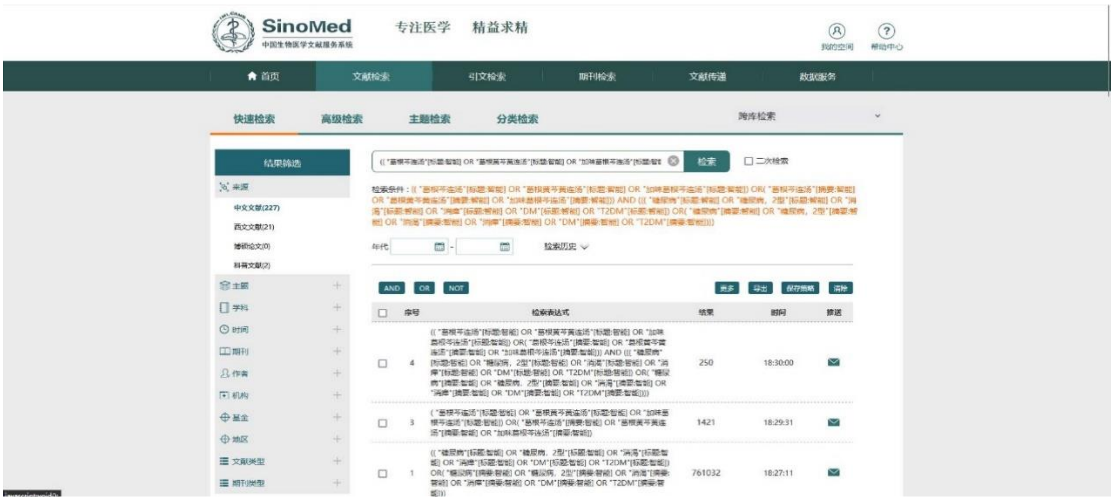

ClinicalTrials

The retrieval of the ClinicalTrials database contained 0 ongoing experiment.

ClinicalTrials.gov — About This Site | Find Studies | Data About Studies | Study Basics | PRS Info

Home > Search Results

The U.S. government does not review or approve the safety and science of all studies listed on this website.
Read our full disclaimer for details.

Focus Your Search (all filters optional)

Condition or disease: Type 2 Diabetes Mellitus

Other terms:

Intervention/Treatment: gegen qinlian

Search Results

Card View | Table View

No results

No records found. Please try different keywords and search again.

For help on searching for studies, see How to Search.

ClinicalTrials.gov — About This Site | Find Studies | Data About Studies | Study Basics | PRS Info

Home > Search Results

The U.S. government does not review or approve the safety and science of all studies listed on this website.
Read our full disclaimer for details.

Focus Your Search (all filters optional)

Condition or disease: Type 2 Diabetes Mellitus

Other terms:

Intervention/Treatment: Gegen Qinlian tang

Search Results

Card View | Table View

No results

No records found. Please try different keywords and search again.

For help on searching for studies, see How to Search.

ClinicalTrials.gov — About This Site | Find Studies | Data About Studies | Study Basics | PRS Info

Home > Search Results

The U.S. government does not review or approve the safety and science of all studies listed on this website.
Read our full disclaimer for details.

Focus Your Search (all filters optional)

Condition or disease: Type 2 Diabetes Mellitus

Other terms:

Intervention/Treatment: Gegen Qinlian decoction

Search Results

Card View | Table View

No results

No records found. Please try different keywords and search again.

For help on searching for studies, see How to Search.

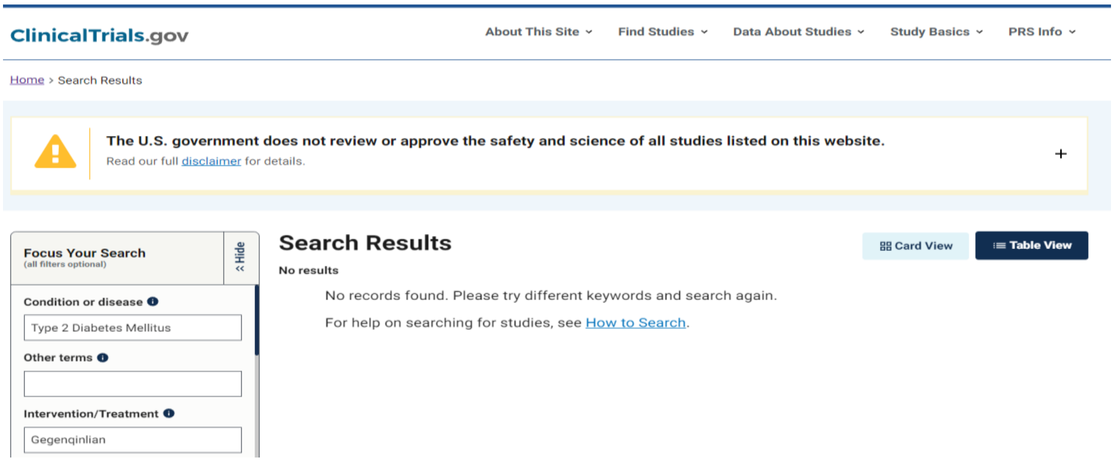

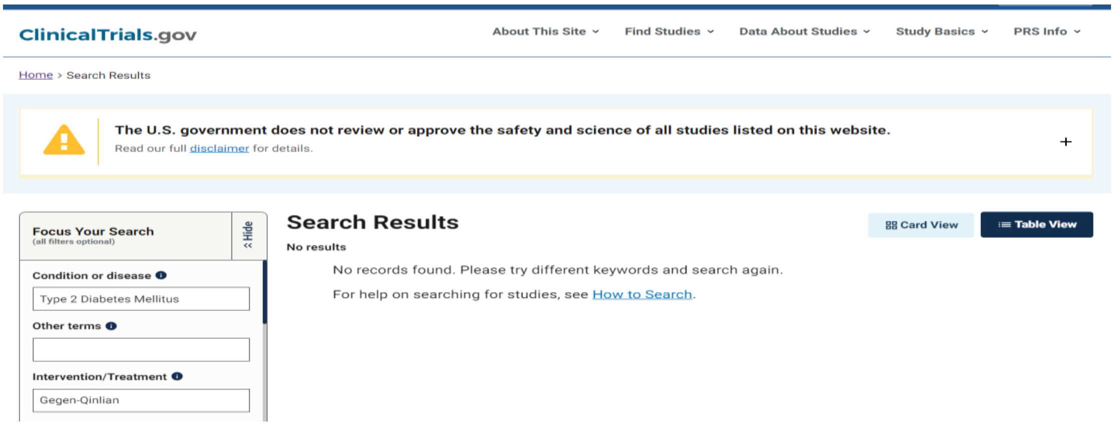

ICTRP

The retrieval of the ICTRP database contained 0 ongoing experiment.

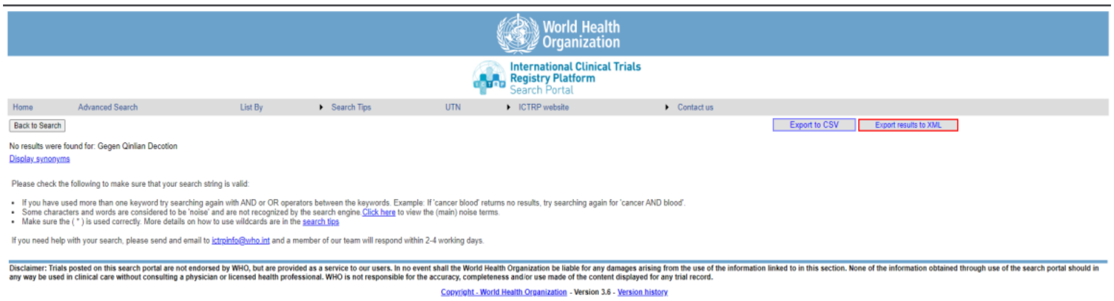

Chinese Clinical Trial Registry

The retrieval of the CHiCTR database contained a total of 1 record. The project has been completed, so there is no ongoing research.

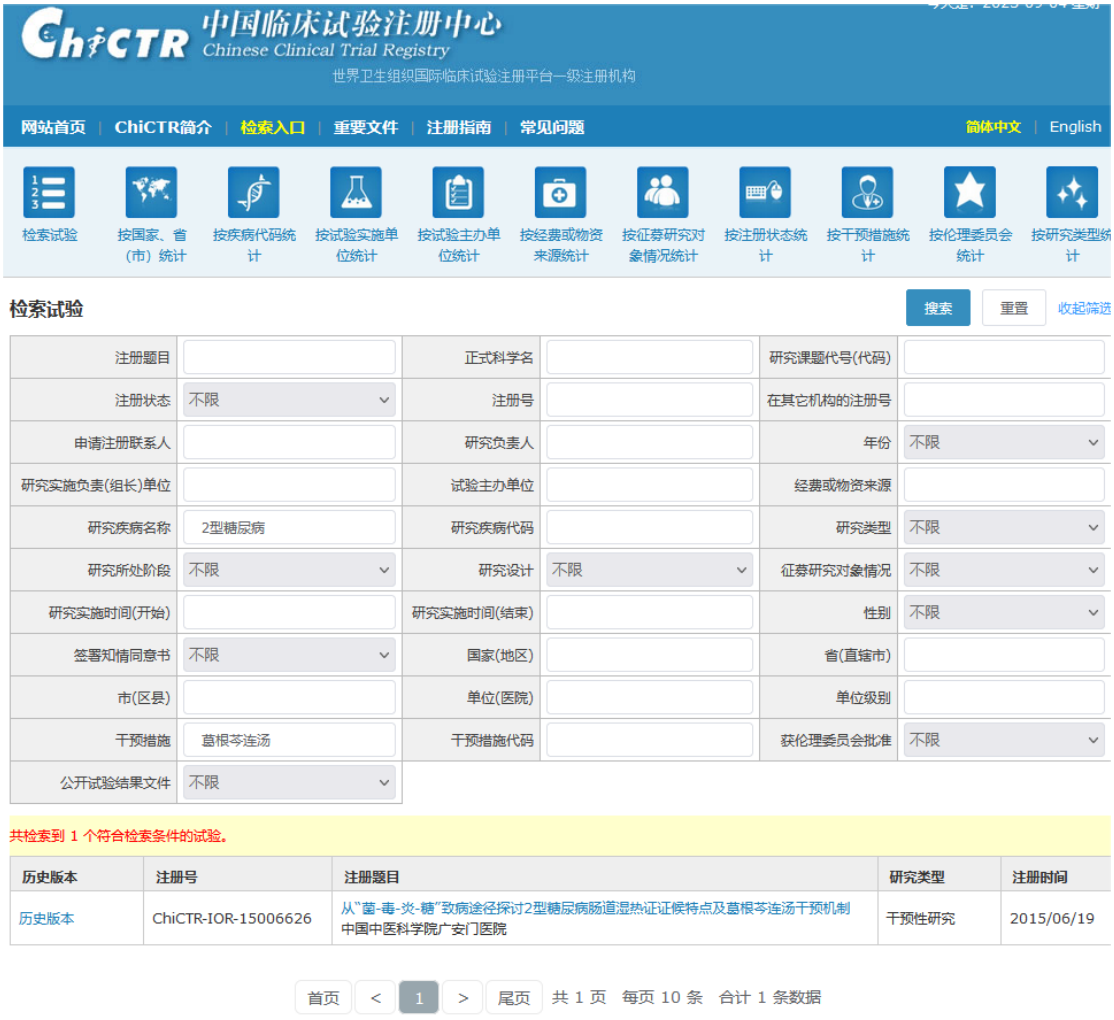

Scopus
TThe retrieval of the CNKI database contained a total of 45 records.

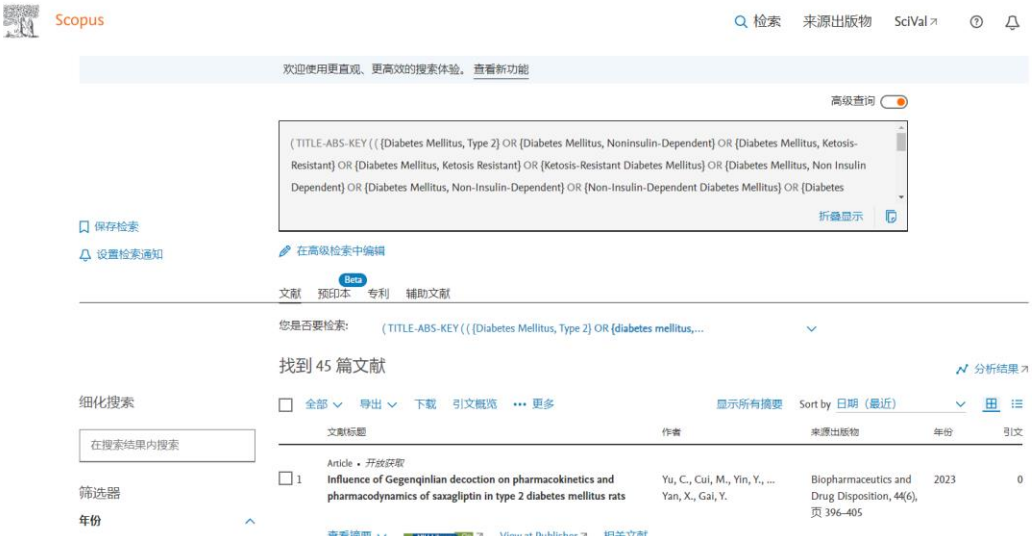

Components of GQD or its modified used in the included studies

Study	Formula	Components
Chen FM (2022)	GQD	Thomson Kudzuvine root (Gegen, Pueraria montana var. thomsonii (Benth.) M.R.Almeida) 15 g, Baical Skullcap Root (Huangqin, Scutellaria baicalensis Georgi) 9 g, Golden thread (Huanglian, Coptis chinensis Franch.) 9 g, Liquorice Root (Gancao, Glycyrrhiza glabra L.) 6 g
Chen XH (2022)	Modified GQD	Thomson Kudzuvine root (Gegen, Pueraria montana var. thomsonii (Benth.) M.R.Almeida) 9 g, Baical Skullcap Root (Huangqin, Scutellaria baicalensis Georgi) 6 g, Golden thread (Huanglian, Coptis chinensis Franch.) 6 g, Rhubarb (Dahuang, Rheum palmatum L.) 6 g, Common Anemarrhena Rhizome (Zhimu, Anemarrhena asphodeloides Bunge) 9 g
Fu YH (2016)	Modified GQD	Thomson Kudzuvine root (Gegen, Pueraria montana var. thomsonii (Benth.) M.R.Almeida) 30 g, Baical Skullcap Root (Huangqin, Scutellaria baicalensis Georgi) 15 g, Golden thread (Huanglian, Coptis chinensis Franch.) 10 g, Liquorice Root (Gancao, Glycyrrhiza glabra L.) 6 g, Tangshen (Dangshen, Codonopsis pilosula (Franch.) Nannf.)30 g, Milkvetch Root (Huangqi, Astragalus mongholicus Bunge) 20 g, Largehead Atractylodes Rh (Baizhu, Atractylodes macrocephala Koidz.) 15 g, Poria cocos（Schw.Wolf.(Fuling, Indian Buead Tuckahoe)20 g, Oriental Water Plantain Rhizome (Zexie, Alisma plantago-aquatica L.)10 g, Polyporusumbellatus(Pers.)Fries(Zhuling, Polyporus)10 g, corn husk(Yumixu)10 g, Cinnamomum cassia Presl.(Guizhi, Cinnamomi Cortex Latin)10 g, Tangshen (Dangshen, Codonopsis pilosula (Franch.) Nannf.)20 g, Dried Ginger (Ganjiang, Zingiber officinale Roscoe) 10 g, Rehmannia glutinosa Libosch root (Shengdi, dried rehmannia root)20 g
Fan YF (2017)	GQD	Thomson Kudzuvine root (Gegen, Pueraria montana var. thomsonii (Benth.) M.R.Almeida) 30 g, Baical Skullcap Root (Huangqin, Scutellaria baicalensis Georgi) 15 g, Golden thread (Huanglian, Coptis chinensis Franch.) 10 g, Liquorice Root (Gancao, Glycyrrhiza glabra L.) 6 g
Gong J (2019)	GQD	Thomson Kudzuvine root (Gegen, Pueraria montana var. thomsonii (Benth.) M.R.Almeida) 30 g, Baical Skullcap Root (Huangqin, Scutellaria baicalensis Georgi) 15 g, Golden thread (Huanglian, Coptis chinensis Franch.) 10 g, Liquorice Root (Gancao, Glycyrrhiza glabra L.) 6 g
Jin J (2019)	GQD	Thomson Kudzuvine root (Gegen, Pueraria montana var. thomsonii (Benth.) M.R.Almeida) 30 g, Baical Skullcap Root (Huangqin, Scutellaria baicalensis Georgi) 20 g, Golden thread (Huanglian, Coptis chinensis Franch.) 6 g, Liquorice Root (Gancao, Glycyrrhiza glabra L.)

		6 g
Li L (2020)	Modified GQD	Thomson Kudzuvine root (Gegen, Pueraria montana var. thomsonii (Benth.) M.R.Almeida) 30 g, Baical Skullcap Root (Huangqin, Scutellaria baicalensis Georgi) 15 g, Liquorice Root (Gancao, Glycyrrhiza glabra L.) 10 g, Tangshen (Dangshen, Codonopsis pilosula (Franch.) Nannf.)30 g, Dried Ginger (Ganjiang, Zingiber officinale Roscoe) 10 g, Largehead Atractylodes Rh (Baizhu, Atractylodes macrocephala Koidz.) 15 g, Baical Skullcap Root (Huangqin, Scutellaria baicalensis Georgi) 20 g, Poria cocos （Schw.Wolf. (Fuling, Indian Buead Tuckahoe)20 g, Danshen Root (Danshen, Salvia miltiorrhiza Bunge) 20 g, Rehmannia glutinosa Libosch root (Shengdi, dried rehmannia root)20 g.
Wang L (2021)	Modified GQD	Thomson Kudzuvine root (Gegen, Pueraria montana var. thomsonii (Benth.) M.R.Almeida) 20 g, Baical Skullcap Root (Huangqin, Scutellaria baicalensis Georgi) 15 g, Golden thread (Huanglian, Coptis chinensis Franch.) 5 g, Liquorice Root (Gancao, Glycyrrhiza glabra L.) 5 g, Snakegourd Fruit (Gualou, Trichosanthes kirilowii Maxim.) 20 g, Danshen Root (Danshen, Salvia miltiorrhiza Bunge) 20 g, Feverwort (Peilan, Eupatoriumfortunei) 15 g, Immature Orange Fruit (Zhishi, Citrus aurantium L) 15 g, Pinellia Tuber [Banxia, Pinellia ternata (Thunb.) Makino] 10 g.
Wang QY (2021)	GQD	Thomson Kudzuvine root (Gegen, Pueraria montana var. thomsonii (Benth.) M.R.Almeida) 25 g, Baical Skullcap Root (Huangqin, Scutellaria baicalensis Georgi) 20 g, Golden thread (Huanglian, Coptis chinensis Franch.) 15 g, Liquorice Root (Gancao, Glycyrrhiza glabra L.) 6 g
Wang Y (2020)	Modified GQD	Thomson Kudzuvine root (Gegen, Pueraria montana var. thomsonii (Benth.) M.R.Almeida) 60 g, Baical Skullcap Root (Huangqin, Scutellaria baicalensis Georgi) 22 g, Golden thread (Huanglian, Coptis chinensis Franch.) 22 g, Liquorice Root (Gancao, Glycyrrhiza glabra L.) 15 g, Dried Ginger (Ganjiang, Zingiber officinale Roscoe) 4 g
Wu L (2021)	Modified GQD	Thomson Kudzuvine root (Gegen, Pueraria montana var. thomsonii (Benth.) M.R.Almeida) 30 g, Baical Skullcap Root (Huangqin, Scutellaria baicalensis Georgi) 15 g, Golden thread (Huanglian, Coptis chinensis Franch.) 15 g, Liquorice Root (Gancao, Glycyrrhiza glabra L.) 6 g, Milkvetch Root (Huangqi, Astragalus mongholicus Bunge) 10g, Dried Ginger (Ganjiang, Zingiber officinale Roscoe) 3 g, Dried orange peel used in Chinese medicine(Chenpi)12 g
Xiong QJ (2019)	Modified GQD	Thomson Kudzuvine root (Gegen, Pueraria montana var. thomsonii (Benth.) M.R.Almeida) 60 g, Baical Skullcap Root (Huangqin, Scutellaria baicalensis Georgi) 22.5 g, Golden thread (Huanglian, Coptis chinensis Franch.) 22.5 g, Liquorice Root (Gancao, Glycyrrhiza glabra L.) 15 g, Dried Ginger (Ganjiang, Zingiber officinale Roscoe)

		3.5 g
Zhang J (2018)	GQD	Thomson Kudzuvine root (Gegen, Pueraria montana var. thomsonii (Benth.) M.R.Almeida) 30 g, Baical Skullcap Root (Huangqin, Scutellaria baicalensis Georgi) 20 g, Golden thread (Huanglian, Coptis chinensis Franch.) 20 g, Liquorice Root (Gancao, Glycyrrhiza glabra L.) 9 g
Zhang LN (2019)	GQD	Thomson Kudzuvine root (Gegen, Pueraria montana var. thomsonii (Benth.) M.R.Almeida) 30 g, Baical Skullcap Root (Huangqin, Scutellaria baicalensis Georgi) 15 g, Golden thread (Huanglian, Coptis chinensis Franch.) 10 g, Liquorice Root (Gancao, Glycyrrhiza glabra L.) 10 g
Zhong XF (2021)	Modified GQD	Thomson Kudzuvine root (Gegen, Pueraria montana var. thomsonii (Benth.) M.R.Almeida) 15 g, Baical Skullcap Root (Huangqin, Scutellaria baicalensis Georgi) 10 g, Golden thread (Huanglian, Coptis chinensis Franch.) 6 g, Liquorice Root (Gancao, Glycyrrhiza glabra L.) 6 g, Rhubarb (Dahuang, Rheum palmatum L.)3 g, Fructus amomi rotundu (Baidoukou, Amomum kravanh Pierre ex Gagnep)10 g, Coix Seed (Yiyiren, Coix lacryma-jobi L.) 20 g, Dried orange peel used in Chinese medicine(Chenpi)10g, Red Peony Root (Chishao, Paeonia lactiflora Pall.) 15 g, Common Anemarrhena Rhizome (Zhimu, Anemarrhena asphodeloides Bunge) 9 g, Fructus Ligustri Lucidi(Nvzhenzi, Ligustrum lucidum Ait) 15 g.
Zhou A (2012)	GQD	No specific dosage was mentioned
Zhou XY (2020)	GQD	Thomson Kudzuvine root (Gegen, Pueraria montana var. thomsonii (Benth.) M.R.Almeida) 24 g, Baical Skullcap Root (Huangqin, Scutellaria baicalensis Georgi) 9 g, Golden thread (Huanglian, Coptis chinensis Franch.) 9 g, Liquorice Root (Gancao, Glycyrrhiza glabra L.) 6 g

Meta-regression analysis of FBG and HbA1c
FBG

1. The results of meta-regression analysis of FBG on average age

```
. meta regress average_age, random(reml) se(khartung)

  Effect-size label: Mean diff.
        Effect size: _meta_es
          Std. err.: _meta_se

Random-effects meta-regression                    Number of obs  =        13
Method: REML                                      Residual heterogeneity:
SE adjustment: Knapp-Hartung                                  tau2 =  .08147
                                                            I2 (%) =   78.60
                                                                H2 =    4.67
                                                     R-squared (%) =    0.00
                                                  Model F(1,11)  =      0.30
                                                  Prob > F       =    0.5975

------------------------------------------------------------------------------
    _meta_es | Coefficient  Std. err.      t    P>|t|     [95% conf. interval]
-------------+----------------------------------------------------------------
 average_age |   -.006749   .0124128    -0.54   0.597    -.0340694    .0205713
       _cons |  -.3406176   .6866644    -0.50   0.630    -1.851956    1.170721
------------------------------------------------------------------------------
Test of residual homogeneity: Q_res = chi2(11) = 36.04   Prob > Q_res = 0.0002
```

2. The results of meta-regression analysis of FBG on sample size.

```
.  meta regress sample_size, random(reml) se(khartung)

  Effect-size label: Mean diff.
        Effect size: _meta_es
          Std. err.: _meta_se

Random-effects meta-regression                    Number of obs  =        13
Method: REML                                      Residual heterogeneity:
SE adjustment: Knapp-Hartung                                  tau2 =  .07612
                                                            I2 (%) =   78.31
                                                                H2 =    4.61
                                                     R-squared (%) =    0.00
                                                  Model F(1,11)  =      0.00
                                                  Prob > F       =    0.9707

------------------------------------------------------------------------------
    _meta_es | Coefficient  Std. err.      t    P>|t|     [95% conf. interval]
-------------+----------------------------------------------------------------
 sample_size |   .0001283   .0034164     0.04   0.971    -.0073913    .0076478
       _cons |  -.7194106   .3227326    -2.23   0.048     -1.42974   -.0090809
------------------------------------------------------------------------------
Test of residual homogeneity: Q_res = chi2(11) = 33.99   Prob > Q_res = 0.0004
```

3. The results of meta-regression analysis of FBG on publication year.

```
. meta regress Year, random(reml) se(khartung)

  Effect-size label: Mean diff.
        Effect size: _meta_es
          Std. err.: _meta_se

Random-effects meta-regression                  Number of obs    =         13
Method: REML                                    Residual heterogeneity:
SE adjustment: Knapp-Hartung                                tau2 =     .07169
                                                          I2 (%) =      77.13
                                                              H2 =       4.37
                                                  R-squared (%) =       0.00
                                                Model F(1,11)    =       0.01
                                                Prob > F         =     0.9341

------------------------------------------------------------------------------
    _meta_es | Coefficient  Std. err.      t    P>|t|     [95% conf. interval]
-------------+----------------------------------------------------------------
        Year |    .005526   .0652995     0.08   0.934    -.1381974    .1492493
       _cons |  -11.86949   131.9112    -0.09   0.930     -302.204     278.465
------------------------------------------------------------------------------
Test of residual homogeneity: Q_res = chi2(11) = 32.92   Prob > Q_res = 0.0005
```

HbA1c

1.The results of meta-regression analysis of HbA1c on average age.

```
. meta regress average_age, random(reml) se(khartung)

  Effect-size label: Mean diff.
        Effect size: _meta_es
          Std. err.: _meta_se

Random-effects meta-regression                  Number of obs    =         13
Method: REML                                    Residual heterogeneity:
SE adjustment: Knapp-Hartung                                tau2 =     .05121
                                                          I2 (%) =      77.88
                                                              H2 =       4.52
                                                  R-squared (%) =       0.00
                                                Model F(1,11)    =       0.06
                                                Prob > F         =     0.8150

------------------------------------------------------------------------------
    _meta_es | Coefficient  Std. err.      t    P>|t|     [95% conf. interval]
-------------+----------------------------------------------------------------
 average_age |  -.0019529   .0081497    -0.24   0.815    -.0198903    .0159846
       _cons |  -.5361624   .4648187    -1.15   0.273    -1.559221    .4868966
------------------------------------------------------------------------------
Test of residual homogeneity: Q_res = chi2(11) = 40.87   Prob > Q_res = 0.0000
```

2. The results of meta-regression analysis of HbA1c on sample size.

```
. meta regress sample_size, random(reml) se(khartung)

  Effect-size label: Mean diff.
        Effect size: _meta_es
          Std. err.: _meta_se

Random-effects meta-regression                    Number of obs   =         13
Method: REML                                      Residual heterogeneity:
SE adjustment: Knapp-Hartung                                  tau2 =   .05027
                                                            I2 (%) =    77.00
                                                                H2 =     4.35
                                                     R-squared (%) =     0.00
                                                  Model F(1,11)   =     0.22
                                                  Prob > F        =   0.6506

------------------------------------------------------------------------------
    _meta_es | Coefficient  Std. err.      t    P>|t|     [95% conf. interval]
-------------+----------------------------------------------------------------
 sample_size |   -.001028   .0022083    -0.47   0.651    -.0058885    .0038325
       _cons |  -.5582477   .2034171    -2.74   0.019    -1.005966   -.1105296
------------------------------------------------------------------------------
Test of residual homogeneity: Q_res = chi2(11) = 40.81   Prob > Q_res = 0.0000
```

3. The results of meta-regression analysis of HbA1c on publication year.

```
. meta regress Year, random(reml) se(khartung)

  Effect-size label: Mean diff.
        Effect size: _meta_es
          Std. err.: _meta_se

Random-effects meta-regression                    Number of obs   =         13
Method: REML                                      Residual heterogeneity:
SE adjustment: Knapp-Hartung                                  tau2 =   .03309
                                                            I2 (%) =    70.07
                                                                H2 =     3.34
                                                     R-squared (%) =    23.71
                                                  Model F(1,11)   =     3.96
                                                  Prob > F        =   0.0719

------------------------------------------------------------------------------
    _meta_es | Coefficient  Std. err.      t    P>|t|     [95% conf. interval]
-------------+----------------------------------------------------------------
        Year |  -.0858407   .0431163    -1.99   0.072    -.1807391    .0090576
       _cons |   172.7645   87.10482     1.98   0.073    -18.95191    364.4809
------------------------------------------------------------------------------
Test of residual homogeneity: Q_res = chi2(11) = 32.91   Prob > Q_res = 0.0005
```

Subgroup analysis

Subgroup analysis of FBG for GQD combined with conventional treatment vs. conventional treatment

1.Average age

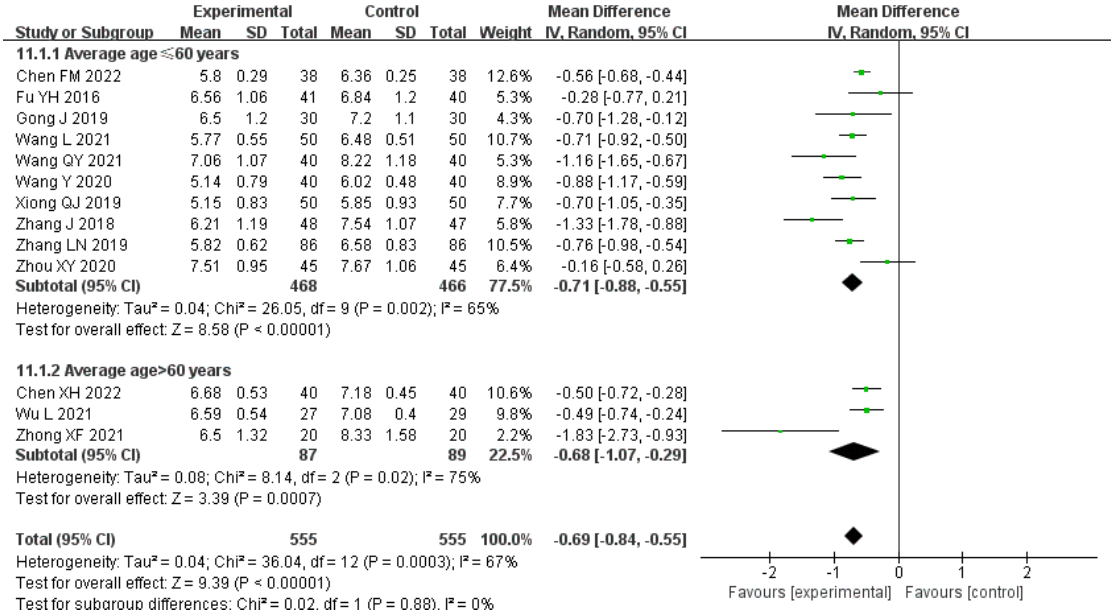

2.Course of disease

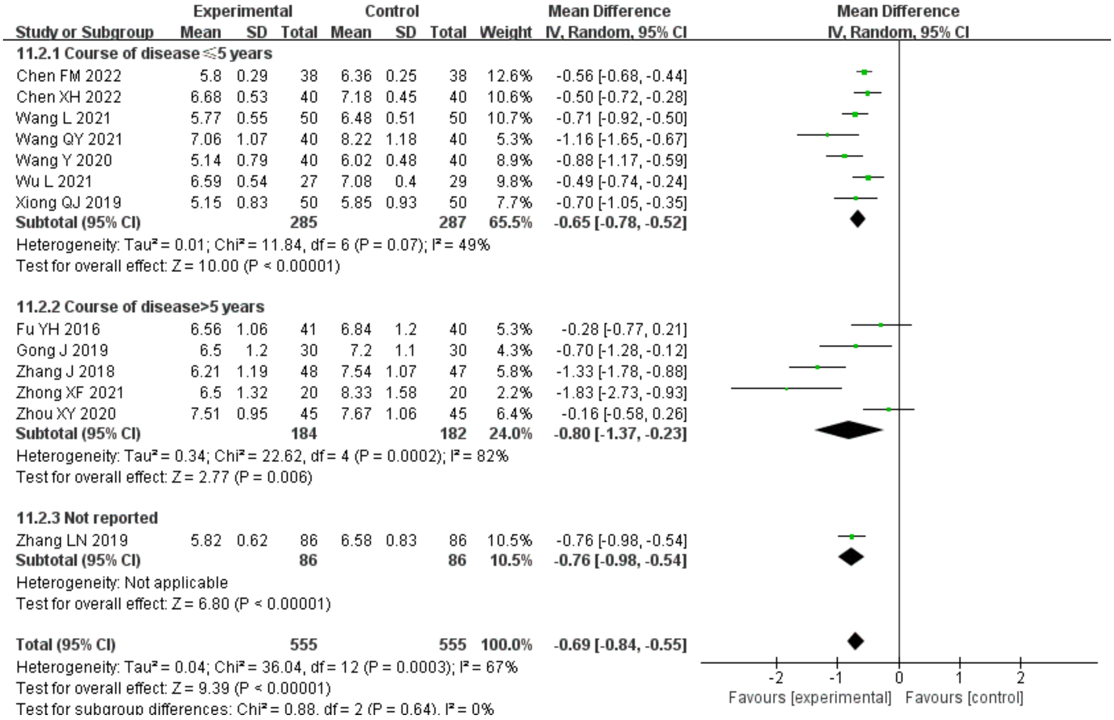

3.Treatment duration

Study or Subgroup	Experimental Mean	Experimental SD	Experimental Total	Control Mean	Control SD	Control Total	Weight	Mean Difference IV, Random, 95% CI
**11.3.1 Treatment duration ≤2 months**								
Chen FM 2022	5.8	0.29	38	6.36	0.25	38	12.6%	-0.56 [-0.68, -0.44]
Fu YH 2016	6.56	1.06	41	6.84	1.2	40	5.3%	-0.28 [-0.77, 0.21]
Gong J 2019	6.5	1.2	30	7.2	1.1	30	4.3%	-0.70 [-1.28, -0.12]
Wang Y 2020	5.14	0.79	40	6.02	0.48	40	8.9%	-0.88 [-1.17, -0.59]
Wu L 2021	6.59	0.54	27	7.08	0.4	29	9.8%	-0.49 [-0.74, -0.24]
Xiong QJ 2019	5.15	0.83	50	5.85	0.93	50	7.7%	-0.70 [-1.05, -0.35]
Zhang J 2018	6.21	1.19	48	7.54	1.07	47	5.8%	-1.33 [-1.78, -0.88]
Zhang LN 2019	5.82	0.62	86	6.58	0.83	86	10.5%	-0.76 [-0.98, -0.54]
**Subtotal (95% CI)**			**360**			**360**	**64.8%**	**-0.69 [-0.86, -0.53]**
Heterogeneity: Tau² = 0.03; Chi² = 17.93, df = 7 (P = 0.01); I² = 61%								
Test for overall effect: Z = 8.31 (P < 0.00001)								
**11.3.2 Treatment duration >2 months**								
Chen XH 2022	6.68	0.53	40	7.18	0.45	40	10.6%	-0.50 [-0.72, -0.28]
Wang L 2021	5.77	0.55	50	6.48	0.51	50	10.7%	-0.71 [-0.92, -0.50]
Wang QY 2021	7.06	1.07	40	8.22	1.18	40	5.3%	-1.16 [-1.65, -0.67]
Zhong XF 2021	6.5	1.32	20	8.33	1.58	20	2.2%	-1.83 [-2.73, -0.93]
Zhou XY 2020	7.51	0.95	45	7.67	1.06	45	6.4%	-0.16 [-0.58, 0.26]
**Subtotal (95% CI)**			**195**			**195**	**35.2%**	**-0.73 [-1.06, -0.39]**
Heterogeneity: Tau² = 0.10; Chi² = 18.10, df = 4 (P = 0.001); I² = 78%								
Test for overall effect: Z = 4.27 (P < 0.0001)								
**Total (95% CI)**			**555**			**555**	**100.0%**	**-0.69 [-0.84, -0.55]**
Heterogeneity: Tau² = 0.04; Chi² = 36.04, df = 12 (P = 0.0003); I² = 67%								
Test for overall effect: Z = 9.39 (P < 0.00001)								
Test for subgroup differences: Chi² = 0.03, df = 1 (P = 0.85), I² = 0%								

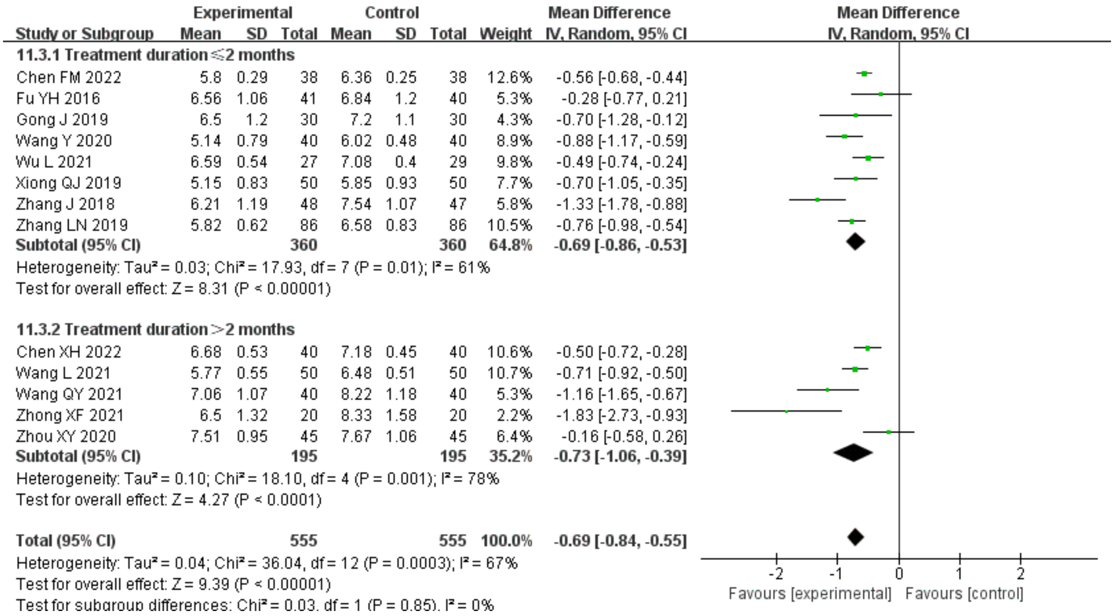

Subgroup analysis of FBG for GQD vs. conventional treatment

1.Course of disease

Study or Subgroup	Experimental Mean	Experimental SD	Experimental Total	Control Mean	Control SD	Control Total	Weight	Mean Difference IV, Fixed, 95% CI
**15.3.1 Course of disease ≤5years**								
Fan YF 2017	5.3	1.12	35	5.86	1.03	35	45.2%	-0.56 [-1.06, -0.06]
Li L 2020	6.43	1.38	44	7.26	1.05	44	43.8%	-0.83 [-1.34, -0.32]
**Subtotal (95% CI)**			**79**			**79**	**89.1%**	**-0.69 [-1.05, -0.33]**
Heterogeneity: Chi² = 0.54, df = 1 (P = 0.46); I² = 0%								
Test for overall effect: Z = 3.78 (P = 0.0002)								
**15.3.2 Not reported**								
Jin J 2019	7.39	2.93	55	8.2	2.54	55	10.9%	-0.81 [-1.83, 0.21]
**Subtotal (95% CI)**			**55**			**55**	**10.9%**	**-0.81 [-1.83, 0.21]**
Heterogeneity: Not applicable								
Test for overall effect: Z = 1.55 (P = 0.12)								
**Total (95% CI)**			**134**			**134**	**100.0%**	**-0.71 [-1.04, -0.37]**
Heterogeneity: Chi² = 0.59, df = 2 (P = 0.75); I² = 0%								
Test for overall effect: Z = 4.08 (P < 0.0001)								
Test for subgroup differences: Chi² = 0.04, df = 1 (P = 0.83), I² = 0%								

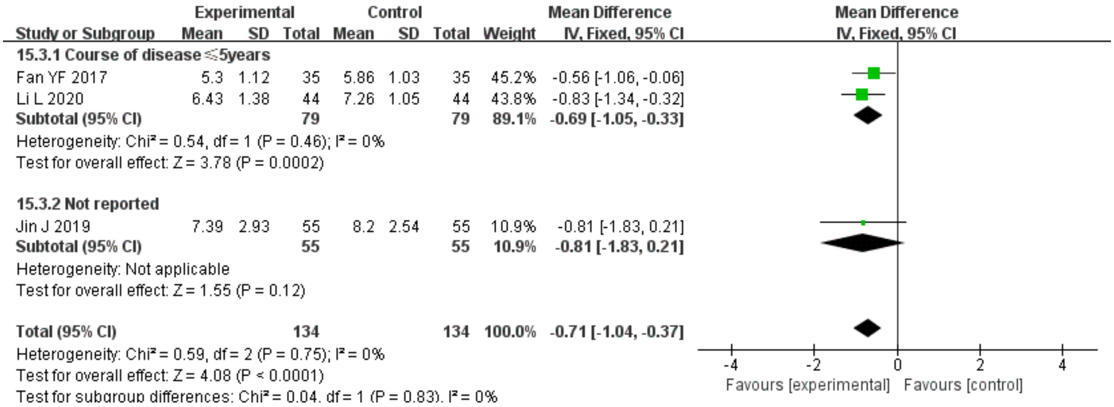

2.Treatment duration

Study or Subgroup	Experimental Mean	Experimental SD	Experimental Total	Control Mean	Control SD	Control Total	Weight	Mean Difference IV, Fixed, 95% CI
**15.2.1 Treatment ≤2months**								
Fan YF 2017	5.3	1.12	35	5.86	1.03	35	45.2%	-0.56 [-1.06, -0.06]
Jin J 2019	7.39	2.93	55	8.2	2.54	55	10.9%	-0.81 [-1.83, 0.21]
**Subtotal (95% CI)**			**90**			**90**	**56.2%**	**-0.61 [-1.06, -0.16]**
Heterogeneity: Chi² = 0.18, df = 1 (P = 0.67); I² = 0%								
Test for overall effect: Z = 2.64 (P = 0.008)								
**15.2.2 Treatment>2months**								
Li L 2020	6.43	1.38	44	7.26	1.05	44	43.8%	-0.83 [-1.34, -0.32]
**Subtotal (95% CI)**			**44**			**44**	**43.8%**	**-0.83 [-1.34, -0.32]**
Heterogeneity: Not applicable								
Test for overall effect: Z = 3.18 (P = 0.001)								
**Total (95% CI)**			**134**			**134**	**100.0%**	**-0.71 [-1.04, -0.37]**
Heterogeneity: Chi² = 0.59, df = 2 (P = 0.75); I² = 0%								
Test for overall effect: Z = 4.08 (P < 0.0001)								
Test for subgroup differences: Chi² = 0.40, df = 1 (P = 0.53), I² = 0%								

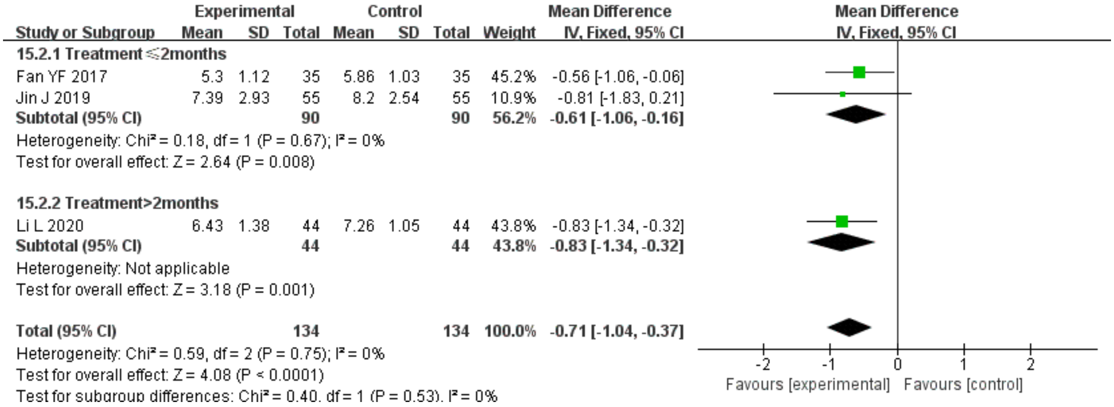

Subgroup analysis of 2hPG for GQD combined with conventional treatment vs. conventional treatment

1. Average age

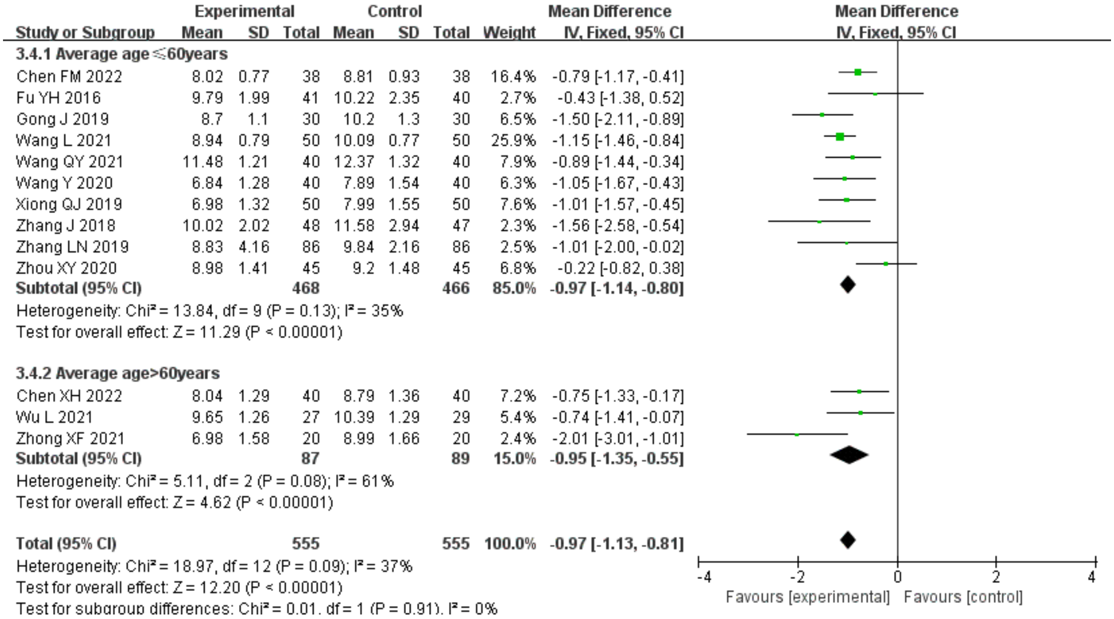

2.Course of disease

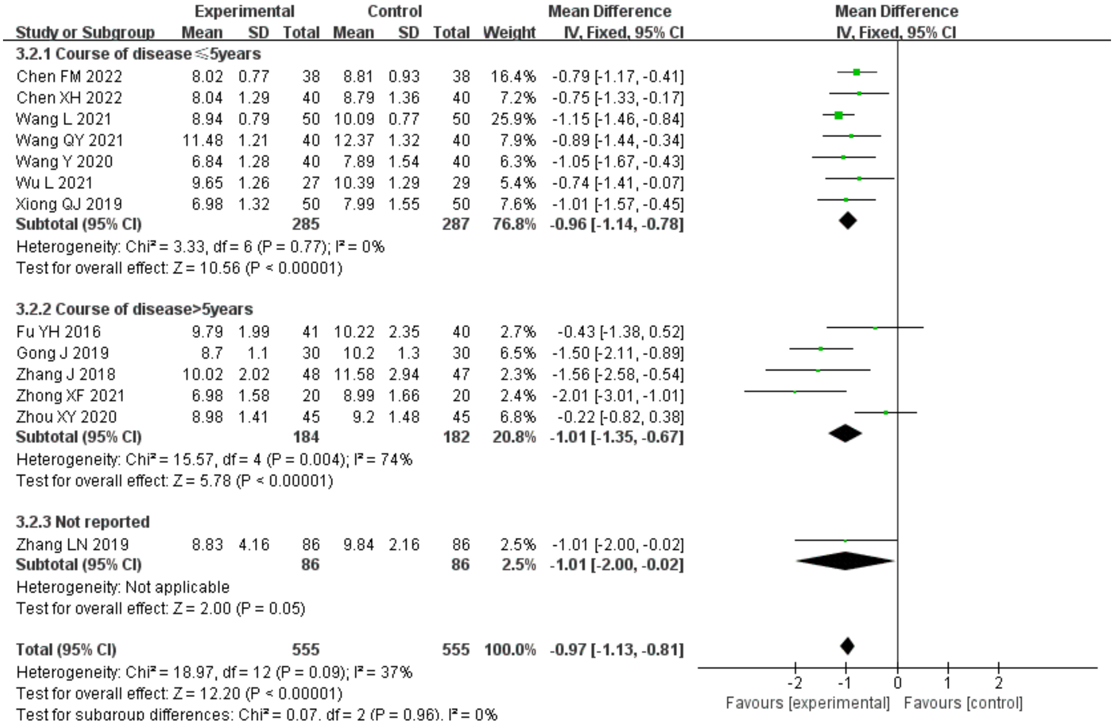

3.Treatment duration

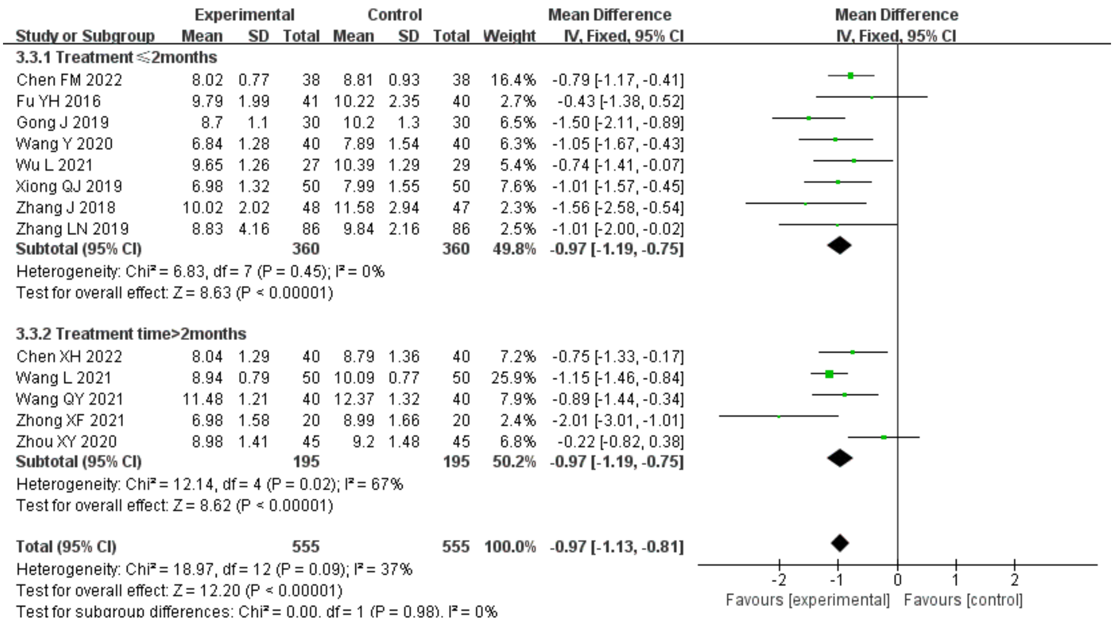

Subgroup analysis of HbA1c for GQD combined with conventional treatment vs. conventional treatment

1.Average age

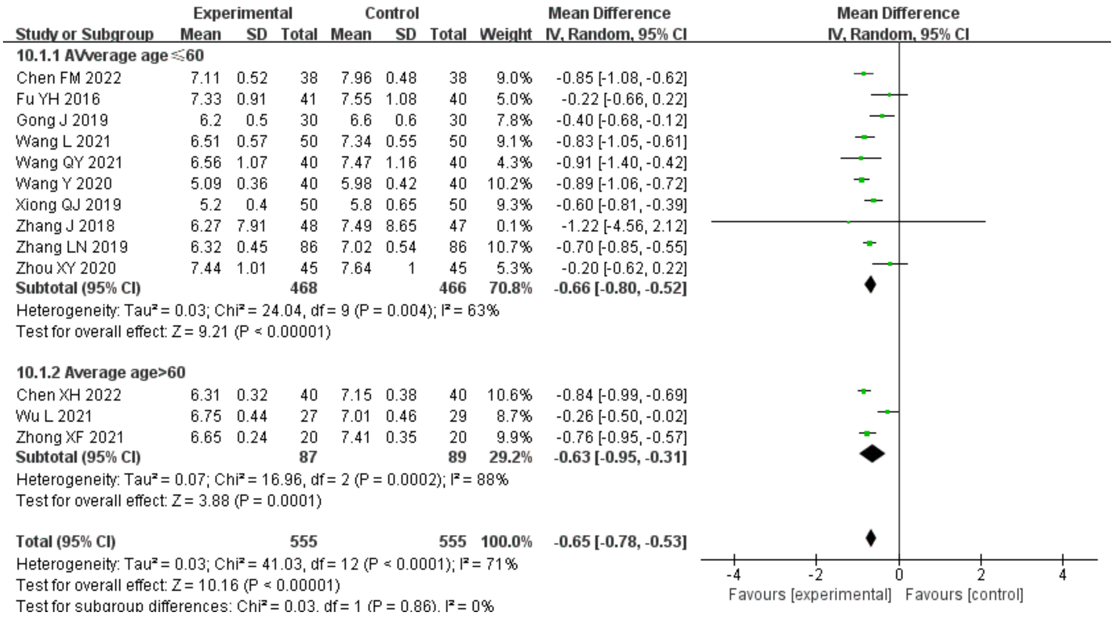

2. Course of disease

Study or Subgroup	Experimental Mean	Experimental SD	Experimental Total	Control Mean	Control SD	Control Total	Weight	Mean Difference IV, Random, 95% CI
**10.2.1 Course of disease≤5 years**								
Chen FM 2022	7.11	0.52	38	7.96	0.48	38	9.0%	-0.85 [-1.08, -0.62]
Chen XH 2022	6.31	0.32	40	7.15	0.38	40	10.6%	-0.84 [-0.99, -0.69]
Wang L 2021	6.51	0.57	50	7.34	0.55	50	9.1%	-0.83 [-1.05, -0.61]
Wang QY 2021	6.56	1.07	40	7.47	1.16	40	4.3%	-0.91 [-1.40, -0.42]
Wang Y 2020	5.09	0.36	40	5.98	0.42	40	10.2%	-0.89 [-1.06, -0.72]
Wu L 2021	6.75	0.44	27	7.01	0.46	29	8.7%	-0.26 [-0.50, -0.02]
Xiong QJ 2019	5.2	0.4	50	5.8	0.65	50	9.3%	-0.60 [-0.81, -0.39]
**Subtotal (95% CI)**			**285**			**287**	**61.2%**	**-0.73 [-0.90, -0.57]**
Heterogeneity: Tau² = 0.04; Chi² = 24.07, df = 6 (P = 0.0005); I² = 75%								
Test for overall effect: Z = 8.69 (P < 0.00001)								
**10.2.2 Course of disease>5 years**								
Fu YH 2016	7.33	0.91	41	7.55	1.08	40	5.0%	-0.22 [-0.66, 0.22]
Gong J 2019	6.2	0.5	30	6.6	0.6	30	7.8%	-0.40 [-0.68, -0.12]
Zhang J 2018	6.27	7.91	48	7.49	8.65	47	0.1%	-1.22 [-4.56, 2.12]
Zhong XF 2021	6.65	0.24	20	7.41	0.35	20	9.9%	-0.76 [-0.95, -0.57]
Zhou XY 2020	7.44	1.01	45	7.64	1	45	5.3%	-0.20 [-0.62, 0.22]
**Subtotal (95% CI)**			**184**			**182**	**28.1%**	**-0.44 [-0.73, -0.16]**
Heterogeneity: Tau² = 0.06; Chi² = 11.08, df = 4 (P = 0.03); I² = 64%								
Test for overall effect: Z = 3.08 (P = 0.002)								
**10.2.3 Not reported**								
Zhang LN 2019	6.32	0.45	86	7.02	0.54	86	10.7%	-0.70 [-0.85, -0.55]
**Subtotal (95% CI)**			**86**			**86**	**10.7%**	**-0.70 [-0.85, -0.55]**
Heterogeneity: Not applicable								
Test for overall effect: Z = 9.24 (P < 0.00001)								
**Total (95% CI)**			**555**			**555**	**100.0%**	**-0.65 [-0.78, -0.53]**
Heterogeneity: Tau² = 0.03; Chi² = 41.03, df = 12 (P < 0.0001); I² = 71%								
Test for overall effect: Z = 10.16 (P < 0.00001)								
Test for subgroup differences: Chi² = 3.16, df = 2 (P = 0.21), I² = 36.7%								

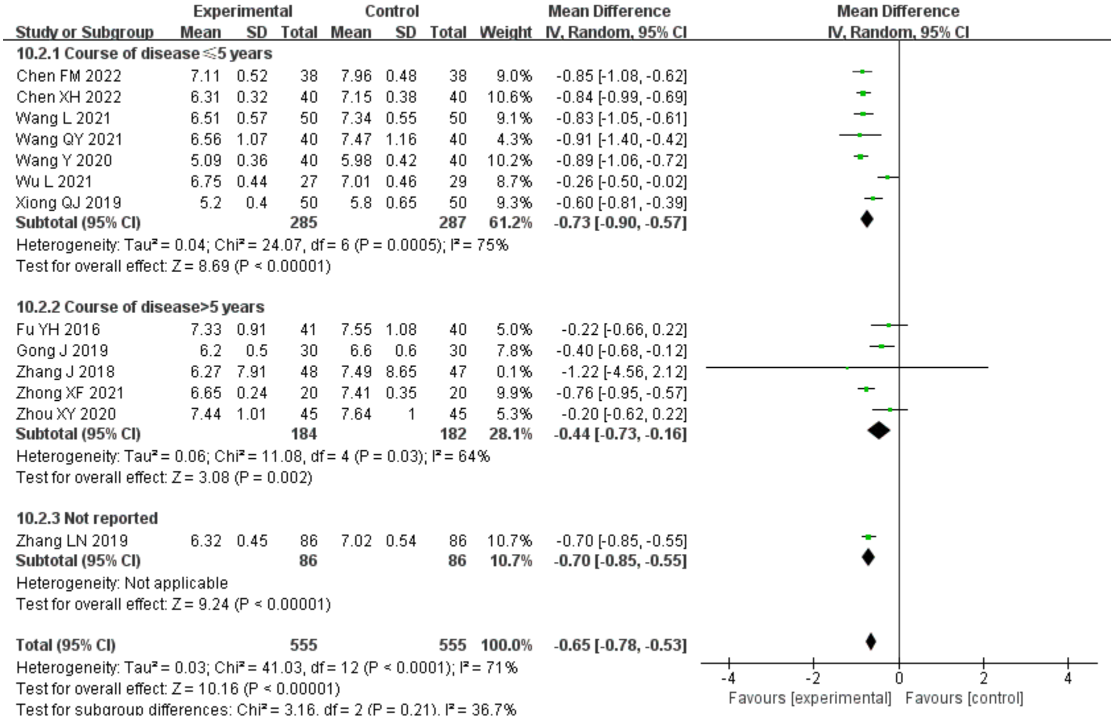

3.Treatment duration

Study or Subgroup	Experimental Mean	Experimental SD	Experimental Total	Control Mean	Control SD	Control Total	Weight	Mean Difference IV, Random, 95% CI
**10.3.1 Treatment duration≤2 months**								
Chen FM 2022	7.11	0.52	38	7.96	0.48	38	9.0%	-0.85 [-1.08, -0.62]
Fu YH 2016	7.33	0.91	41	7.55	1.08	40	5.0%	-0.22 [-0.66, 0.22]
Gong J 2019	6.2	0.5	30	6.6	0.6	30	7.8%	-0.40 [-0.68, -0.12]
Wang Y 2020	5.09	0.36	40	5.98	0.42	40	10.2%	-0.89 [-1.06, -0.72]
Wu L 2021	6.75	0.44	27	7.01	0.46	29	8.7%	-0.26 [-0.50, -0.02]
Xiong QJ 2019	5.2	0.4	50	5.8	0.65	50	9.3%	-0.60 [-0.81, -0.39]
Zhang J 2018	6.27	7.91	48	7.49	8.65	47	0.1%	-1.22 [-4.56, 2.12]
Zhang LN 2019	6.32	0.45	86	7.02	0.54	86	10.7%	-0.70 [-0.85, -0.55]
**Subtotal (95% CI)**			**360**			**360**	**60.8%**	**-0.59 [-0.77, -0.41]**
Heterogeneity: Tau² = 0.04; Chi² = 28.64, df = 7 (P = 0.0002); I² = 76%								
Test for overall effect: Z = 6.47 (P < 0.00001)								
**10.3.2 Treatment duration>2 months**								
Chen XH 2022	6.31	0.32	40	7.15	0.38	40	10.6%	-0.84 [-0.99, -0.69]
Wang L 2021	6.51	0.57	50	7.34	0.55	50	9.1%	-0.83 [-1.05, -0.61]
Wang QY 2021	6.56	1.07	40	7.47	1.16	40	4.3%	-0.91 [-1.40, -0.42]
Zhong XF 2021	6.65	0.24	20	7.41	0.35	20	9.9%	-0.76 [-0.95, -0.57]
Zhou XY 2020	7.44	1.01	45	7.64	1	45	5.3%	-0.20 [-0.62, 0.22]
**Subtotal (95% CI)**			**195**			**195**	**39.2%**	**-0.75 [-0.91, -0.59]**
Heterogeneity: Tau² = 0.02; Chi² = 8.59, df = 4 (P = 0.07); I² = 53%								
Test for overall effect: Z = 9.06 (P < 0.00001)								
**Total (95% CI)**			**555**			**555**	**100.0%**	**-0.65 [-0.78, -0.53]**
Heterogeneity: Tau² = 0.03; Chi² = 41.03, df = 12 (P < 0.0001); I² = 71%								
Test for overall effect: Z = 10.16 (P < 0.00001)								
Test for subgroup differences: Chi² = 1.63, df = 1 (P = 0.20), I² = 38.5%								

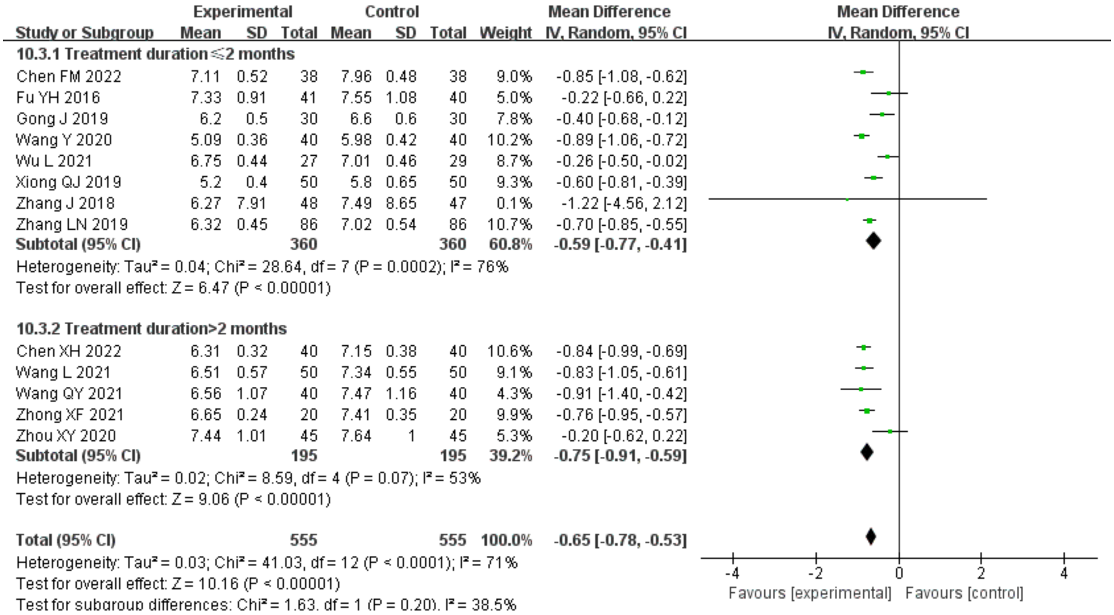

Subgroup analysis of HbA1c for GQD vs. conventional treatment

1.Course of disease

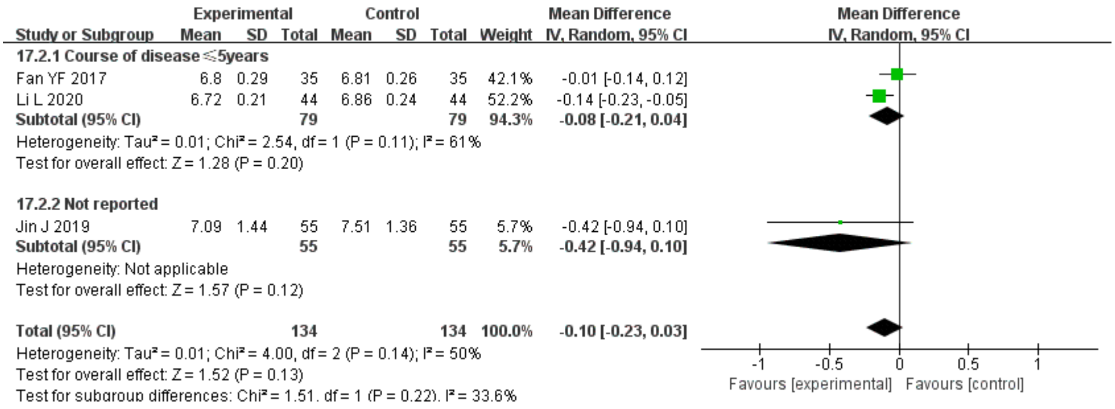

2. Treatment duration

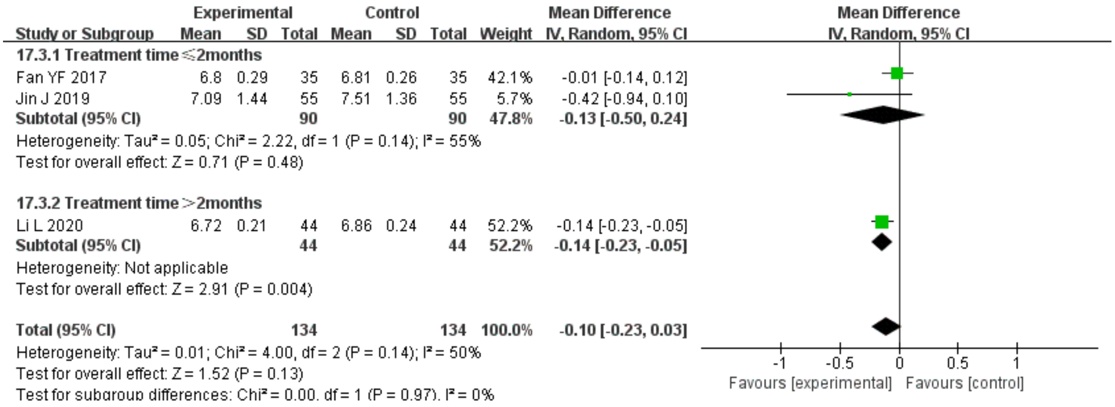

Subgroup analysis of TC for GQD combined with conventional treatment vs. conventional treatment

1.Course of disease

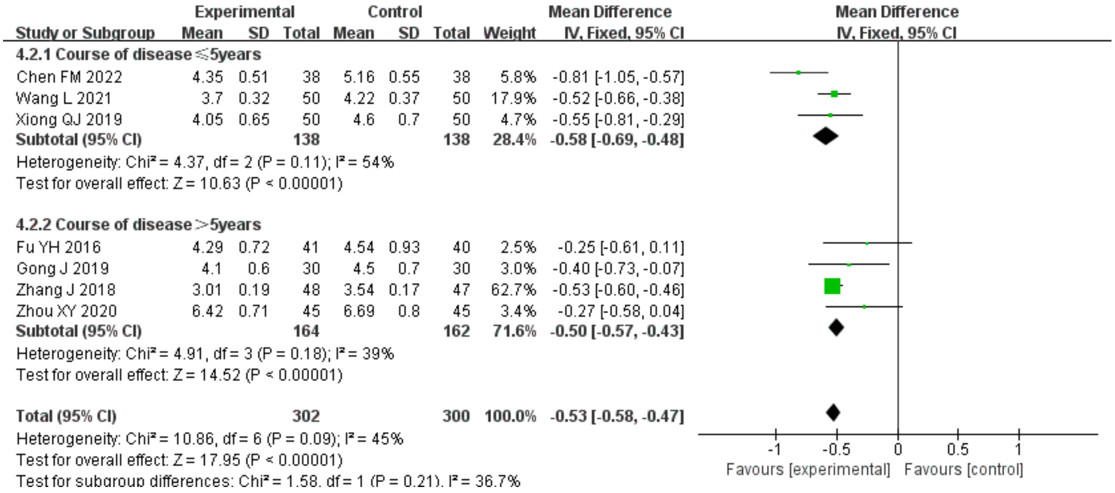

2. Treatment duration

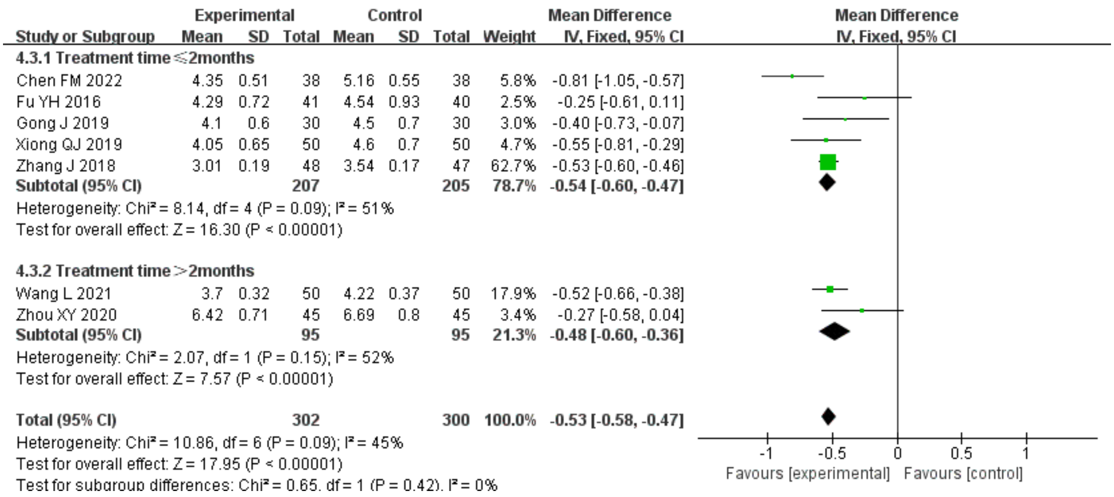

Subgroup analysis of TC for GQD vs. conventional treatment

1.Course of disease

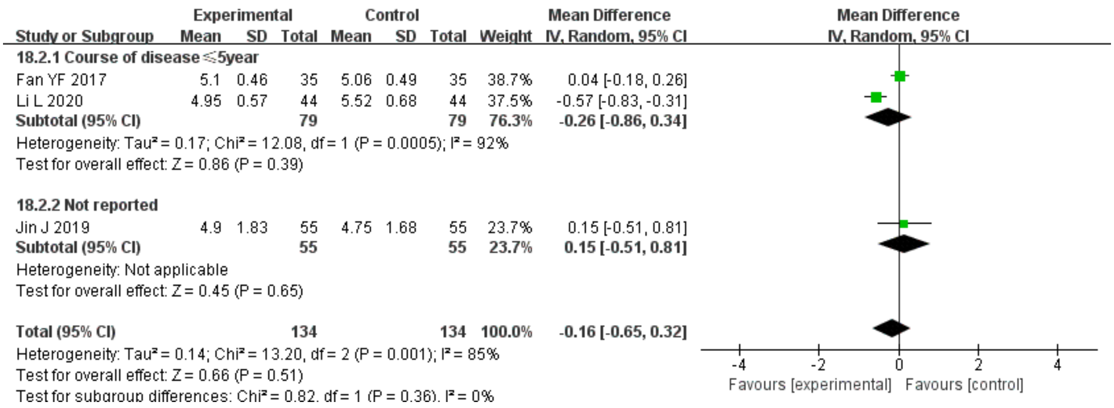

2.Treatment duration

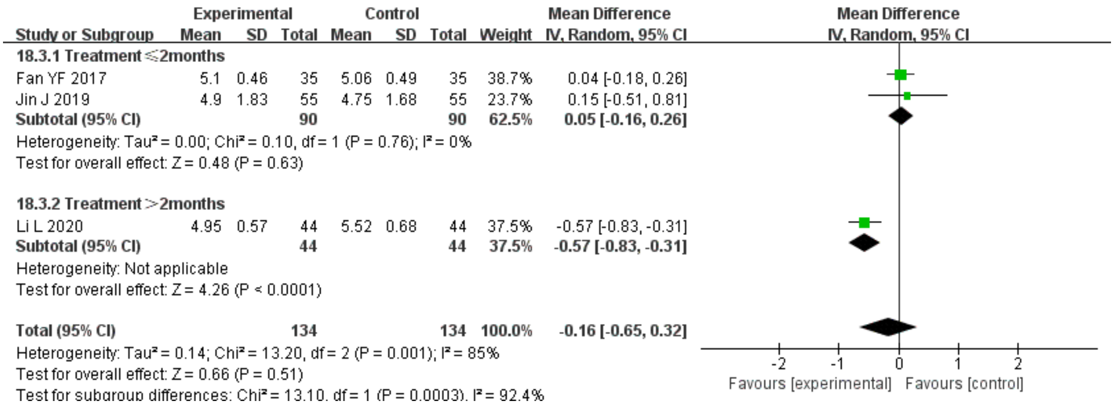

Subgroup analysis of TG for GQD combined with conventional treatment vs. conventional treatment

1. Course of disease

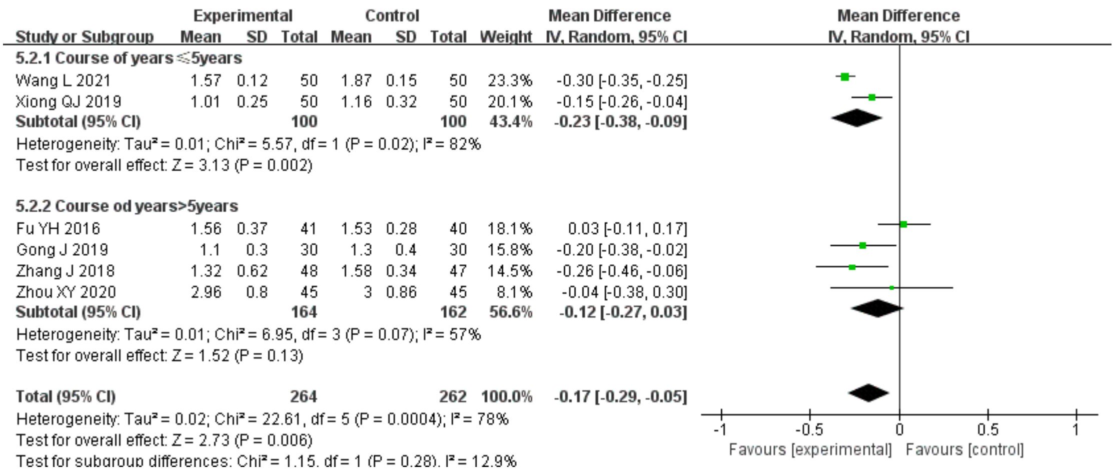

2. Treatment duration

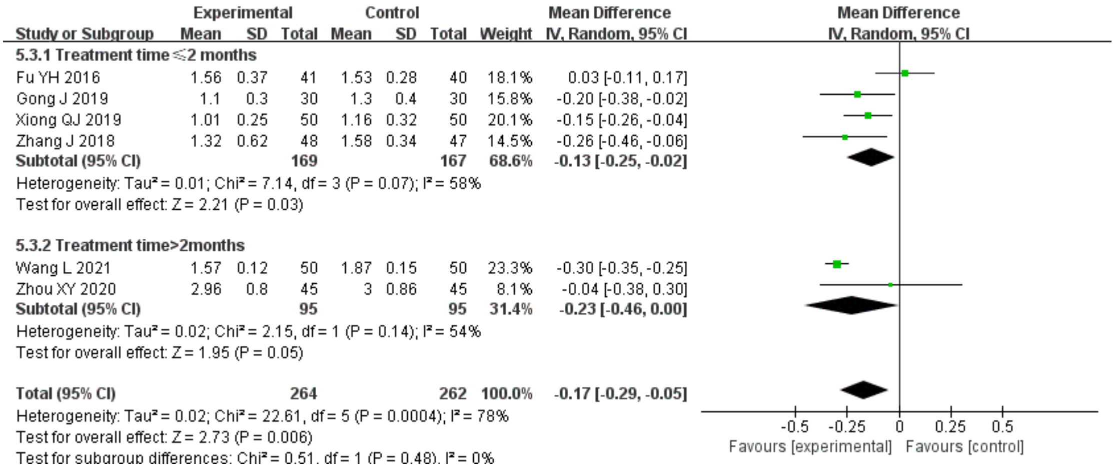

Subgroup analysis of HDL-C for GQD combined with conventional treatment vs. conventional treatment

1. Course of disease

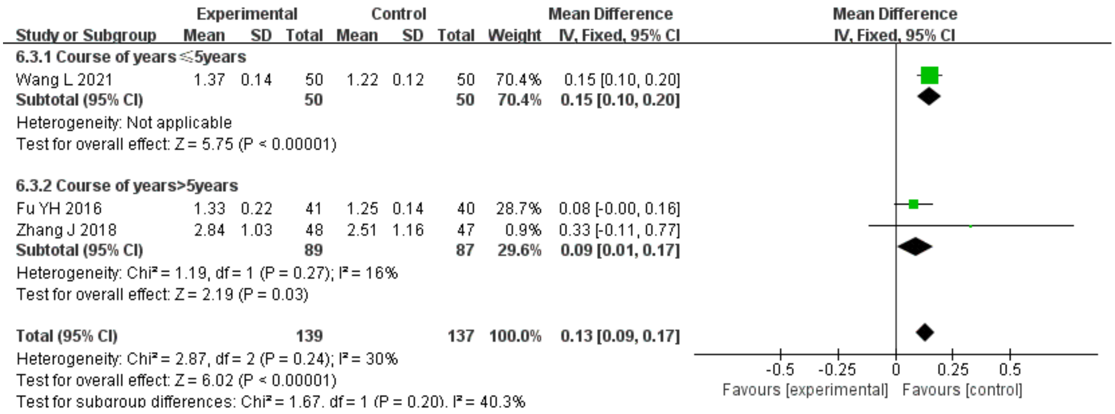

2. Treatment duration

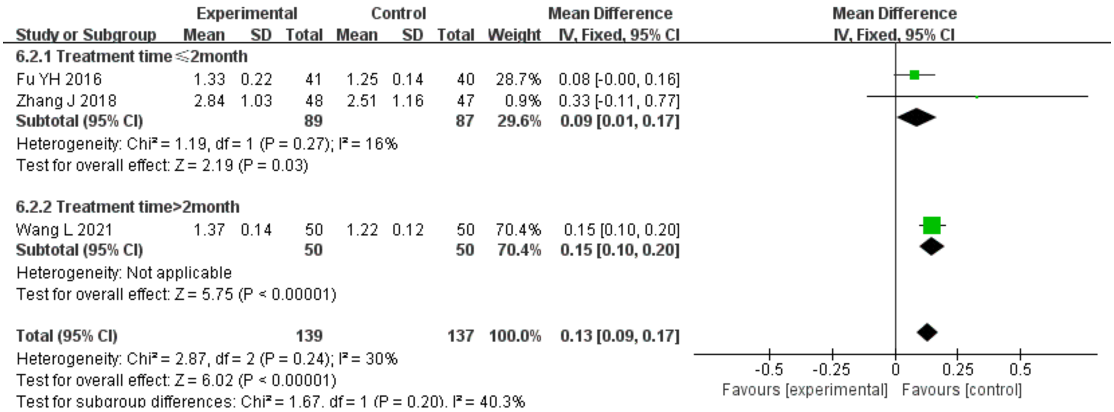

Subgroup analysis of LDL-C for GQD combined with conventional treatment vs. conventional treatment

1. Course of disease

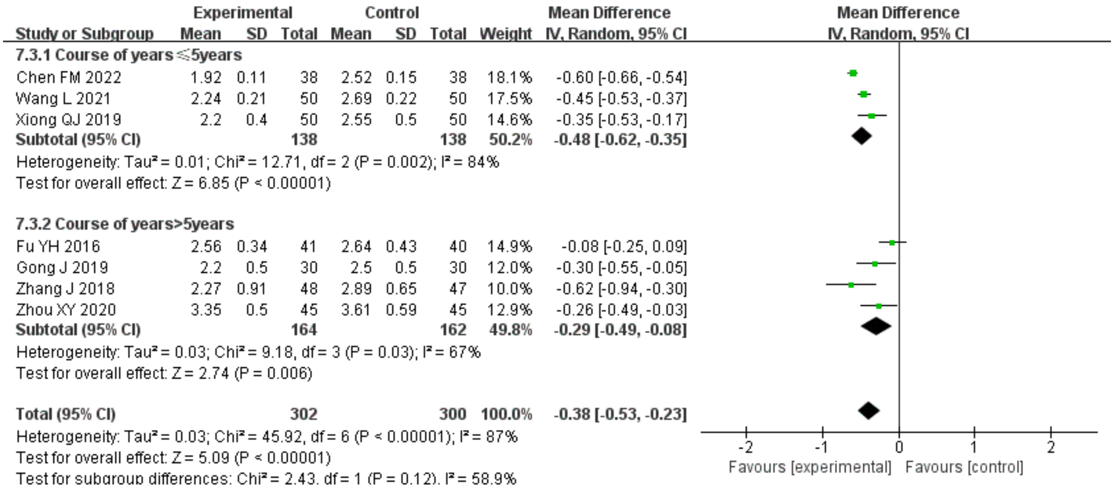

2. Treatment duration

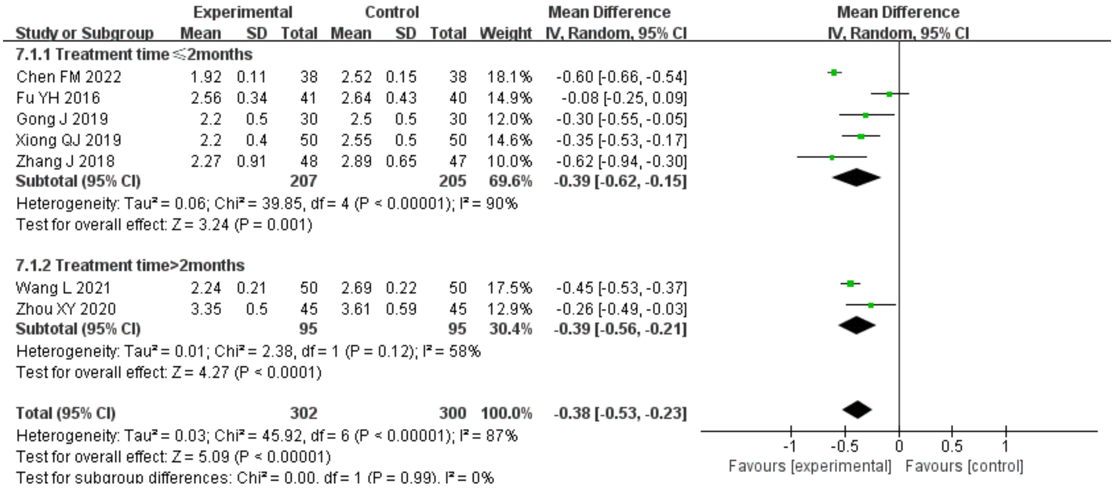

Subgroup analysis of LDL-C for GQD vs. conventional treatment

1.Course of disease

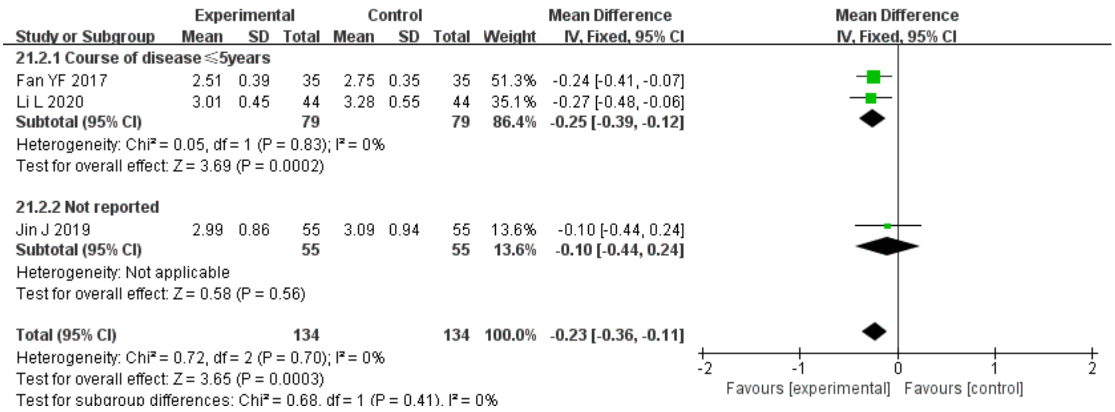

2. Treatment duration

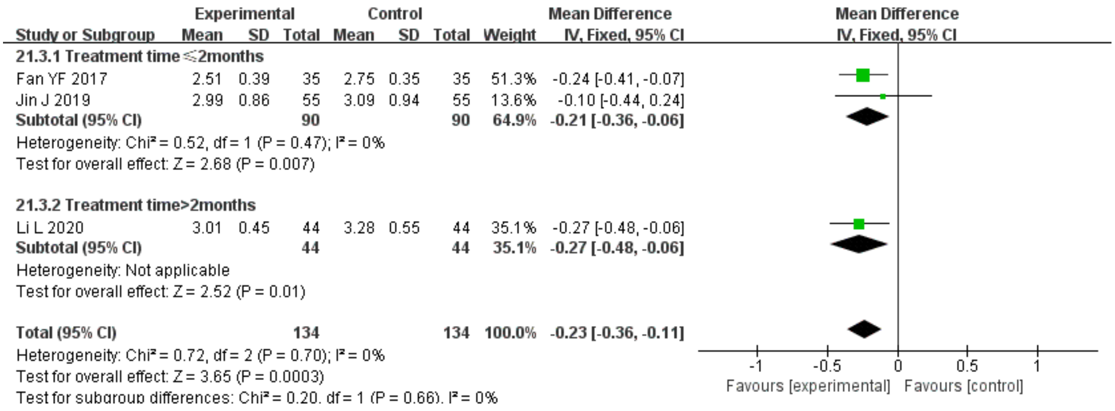

Subgroup analysis of HOMA-IR for GQD combined with conventional treatment vs. conventional treatment

1.Average age

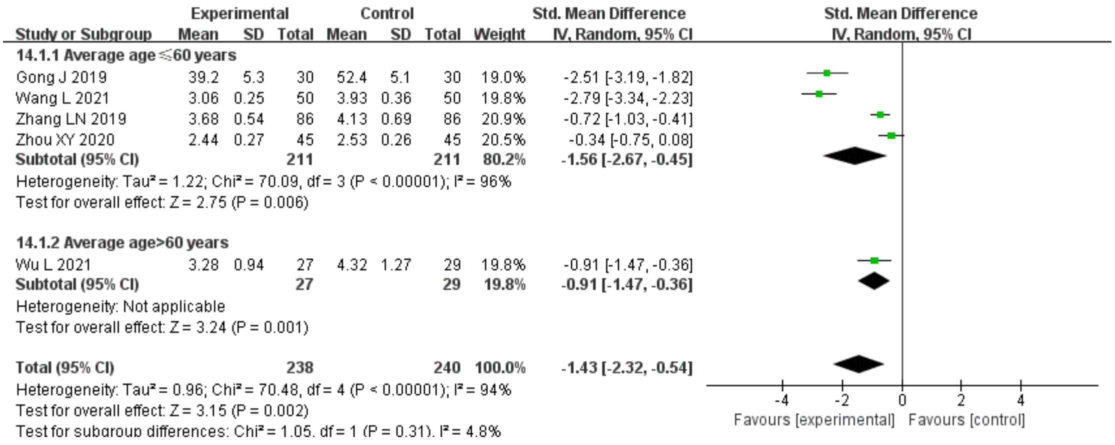

2. Course of disease

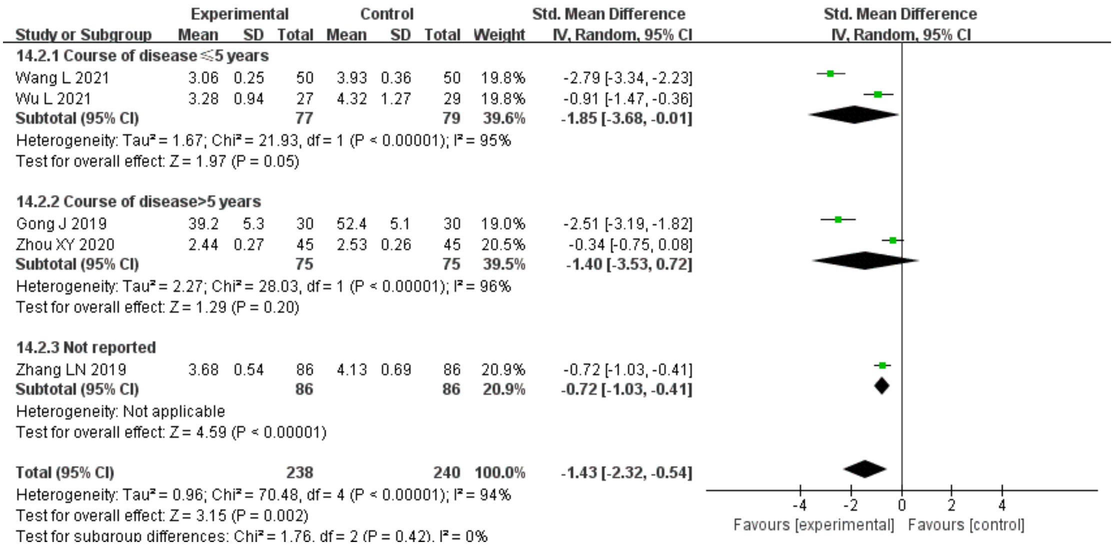

3. Treatment duration

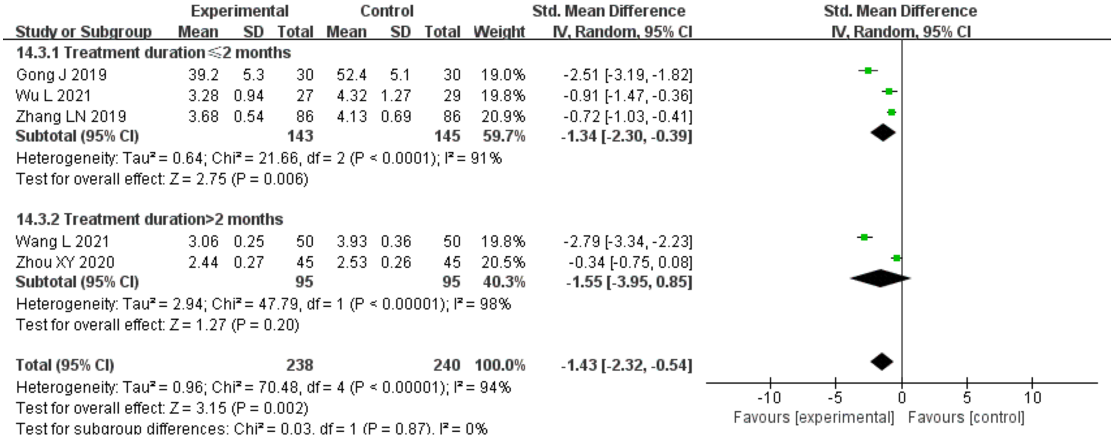

Sensitivity analysis

1.The results of sensitivity analysis of FBG for GQD combined with conventional treatment vs. conventional treatment.

Study omitted	Estimate	[95% Conf.	Interval]
Chen FM (2022)	-.71826226	-.88892978	-.54759479
Chen XH (2022)	-.71829295	-.87740523	-.55918062
Fu YH (2016)	-.71544689	-.86333352	-.56756026
Gong J (2019)	-.69390917	-.84470832	-.54311001
Wang L (2021)	-.69554645	-.85810399	-.53298885
Wang QY (2021)	-.66479778	-.80768549	-.52191007
Wang Y (2020)	-.67459464	-.82646894	-.52272028
Wu L (2021)	-.71700233	-.87410468	-.55990005
Xiong QJ (2019)	-.69457746	-.85000956	-.53914529
Zhang J (2018)	-.6484049	-.7809208	-.51588905
Zhang LN (2019)	-.68856621	-.84831488	-.52881753
Zhong XF (2021)	-.66491377	-.79946262	-.53036493
Zhou XY (2020)	-.7249648	-.86743736	-.58249229
Combined	-.69282233	-.83744075	-.5482039

2. The results of sensitivity analysis of FBG for GQD vs. conventional treatment.

Study omitted	Estimate	[95% Conf.	Interval]
Fan YF (2017)	-.82600039	-1.2842816	-.36771923
Jin J (2019)	-.69280452	-1.0521446	-.33346432
Li L (2020)	-.6087063	-1.061044	-.15636864
Combined	-.70563611	-1.0447342	-.36653798

3. The results of sensitivity analysis of 2hPG for GQD combined with conventional treatment vs. conventional treatment.

Study omitted	Estimate	[95% Conf.	Interval]
Chen FM (2022)	-.98905289	-1.2241516	-.75395423
Chen XH (2022)	-.98336077	-1.2105337	-.75618786
Fu YH (2016)	-.9855836	-1.2005273	-.77063984
Gong J (2019)	-.91741037	-1.1256235	-.70919728
Wang L (2021)	-.93150121	-1.16755	-.69545245
Wang QY (2021)	-.97138005	-1.2029049	-.73985517
Wang Y (2020)	-.95682091	-1.1860657	-.72757614
Wu L (2021)	-.98064947	-1.2058064	-.75549263
Xiong QJ (2019)	-.95982236	-1.1913892	-.72825551
Zhang J (2018)	-.94071382	-1.1546274	-.7268002
Zhang LN (2019)	-.96195215	-1.1845677	-.73933661
Zhong XF (2021)	-.92714506	-1.120474	-.73381615
Zhou XY (2020)	-1.0232333	-1.2020031	-.84446341
(.)	-.96310157	-1.1738775	-.75232565
Combined	-.96310159	-1.1738775	-.75232565

4. The results of sensitivity analysis of HbA1c for GQD combined with conventional treatment vs. conventional treatment.

Study omitted	Estimate	[95% Conf.	Interval]
Chen FM (2022)	-.6328786	-.76820576	-.49755141
Chen XH (2022)	-.62986785	-.76780432	-.49193135
Fu YH (2016)	-.67791295	-.80194116	-.55388469
Gong J (2019)	-.67632192	-.80392009	-.54872376
Wang L (2021)	-.63425696	-.77053112	-.49798286
Wang QY (2021)	-.6412046	-.77150548	-.51090378
Wang Y (2020)	-.62668765	-.75978482	-.49359053
Wu L (2021)	-.70209485	-.81203133	-.59215832
Xiong QJ (2019)	-.6569711	-.79428071	-.51966149
Zhang J (2018)	-.65218723	-.77969909	-.52467537
Zhang LN (2019)	-.64361322	-.78855562	-.49867085
Zhong XF (2021)	-.63901842	-.77930844	-.49872839
Zhou XY (2020)	-.68083745	-.80351472	-.55816013
Combined	-.65356941	-.77958859	-.52755023

5. The results of sensitivity analysis of HbA1c for GQD vs. conventional treatment.

Study omitted	Estimate	[95% Conf.	Interval]
Fan YF (2017)	-.15675078	-.28690228	-.02659928
Jin J (2019)	-.08278047	-.20926216	.04370122
Li L (2020)	-.13329679	-.50179911	.23520553
Combined	-.10125012	-.23177204	.0292718

6. The results of sensitivity analysis of HbA1c for GQD vs. conventional treatment after excluding Fan YF,2017.

Study or Subgroup	Experimental Mean	Experimental SD	Experimental Total	Control Mean	Control SD	Control Total	Weight	Mean Difference IV, Random, 95% CI
Jin J 2019	7.09	1.44	55	7.51	1.36	55	6.0%	-0.42 [-0.94, 0.10]
Li L 2020	6.72	0.21	44	6.86	0.24	44	94.0%	-0.14 [-0.23, -0.05]
**Total (95% CI)**			**99**			**99**	**100.0%**	**-0.16 [-0.29, -0.03]**

Heterogeneity: Tau² = 0.00; Chi² = 1.06, df = 1 (P = 0.30); I² = 6%
Test for overall effect: Z = 2.36 (P = 0.02)

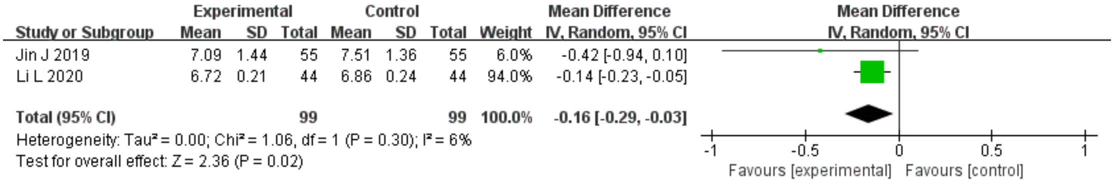

7. The results of sensitivity analysis of TC for GQD combined with conventional treatment vs. conventional treatment.

Study omitted	Estimate	[95% Conf.	Interval]
Chen FM (2022)	-.50703722	-.56785315	-.44622129
Fu YH (2016)	-.533481	-.63325799	-.43370402
Gong J (2019)	-.52276856	-.63468409	-.41085306
Wang L (2021)	-.50334573	-.64478713	-.36190432
Xiong QJ (2019)	-.50640726	-.62498885	-.3878257
Zhang J (2018)	-.49619213	-.65036625	-.34201795
Zhou XY (2020)	-.53717422	-.63555288	-.43879557
Combined	-.51430738	-.61706509	-.41154966

8. The results of sensitivity analysis of TC for GQD combined with conventional treatment vs. conventional treatment after excluding Chen FM,2021.

	Experimental			Control				Mean Difference
Study or Subgroup	Mean	SD	Total	Mean	SD	Total	Weight	IV, Fixed, 95% CI
Fu YH 2016	4.29	0.72	41	4.54	0.93	40	2.7%	-0.25 [-0.61, 0.11]
Gong J 2019	4.1	0.6	30	4.5	0.7	30	3.2%	-0.40 [-0.73, -0.07]
Wang L 2021	3.7	0.32	50	4.22	0.37	50	19.0%	-0.52 [-0.66, -0.38]
Xiong QJ 2019	4.05	0.65	50	4.6	0.7	50	5.0%	-0.55 [-0.81, -0.29]
Zhang J 2018	3.01	0.19	48	3.54	0.17	47	66.6%	-0.53 [-0.60, -0.46]
Zhou XY 2020	6.42	0.71	45	6.69	0.8	45	3.6%	-0.27 [-0.58, 0.04]
**Total (95% CI)**			**264**			**262**	**100.0%**	**-0.51 [-0.57, -0.45]**

Heterogeneity: Chi² = 5.06, df = 5 (P = 0.41); I² = 1%
Test for overall effect: Z = 16.85 (P < 0.00001)

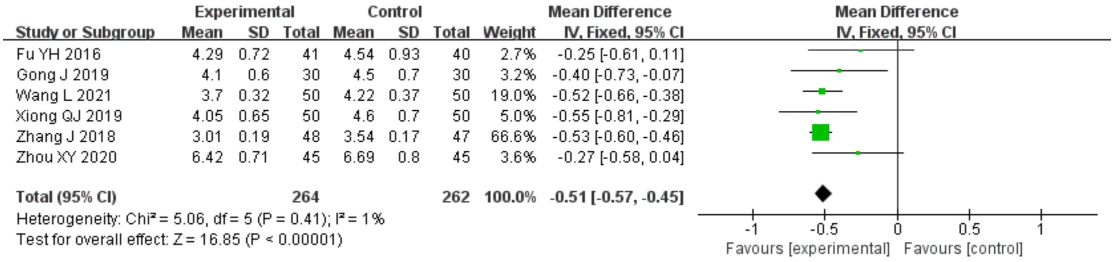

9. The results of sensitivity analysis of TC for GQD vs. conventional treatment.

Study omitted	Estimate	[95% Conf.	Interval]
Fan YF (2017)	-.27549461	-.96930653	.41831726
Jin J (2019)	-.26091191	-.85864723	.3368234
Li L (2020)	.05134694	-.15951493	.26220882
Combined	-.16285887	-.64653442	.32081667

10. The results of sensitivity analysis of TC for GQD vs. conventional treatment after excluding Li L,2020.

	Experimental			Control				Mean Difference
Study or Subgroup	Mean	SD	Total	Mean	SD	Total	Weight	IV, Random, 95% CI
Fan YF 2017	5.1	0.46	35	5.06	0.49	35	89.7%	0.04 [-0.18, 0.26]
Jin J 2019	4.9	1.83	55	4.75	1.68	55	10.3%	0.15 [-0.51, 0.81]
**Total (95% CI)**			**90**			**90**	**100.0%**	**0.05 [-0.16, 0.26]**

Heterogeneity: Tau² = 0.00; Chi² = 0.10, df = 1 (P = 0.76); I² = 0%
Test for overall effect: Z = 0.48 (P = 0.63)

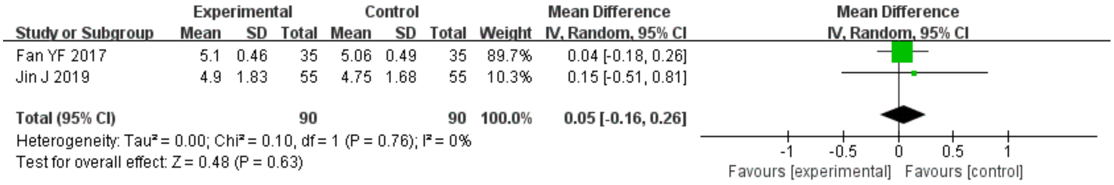

11. The results of sensitivity analysis of TG for GQD combined with conventional treatment vs. conventional treatment.

Study omitted	Estimate	[95% Conf.	Interval]
Fu YH (2016)	-.22511898	-.3125028	-.13773517
Gong J (2019)	-.15901557	-.30061412	-.017417
Wang L (2021)	-.12534083	-.23078707	-.01989459
Xiong QJ (2019)	-.16817854	-.32074311	-.015614
Zhang J (2018)	-.14936343	-.28845981	-.01026704
Zhou XY (2020)	-.17836688	-.30483237	-.05190141
Combined	-.16718138	-.28718675	-.04717602

12. The results of sensitivity analysis of TG for GQD vs. conventional treatment after changing the effect model.

	Experimental			Control				Mean Difference
Study or Subgroup	Mean	SD	Total	Mean	SD	Total	Weight	IV, Random, 95% CI
Jin J 2019	2.81	1.39	55	3.04	1.94	55	2.6%	-0.23 [-0.86, 0.40]
Li L 2020	1.55	0.26	44	1.76	0.23	44	97.4%	-0.21 [-0.31, -0.11]
**Total (95% CI)**			**99**			**99**	**100.0%**	**-0.21 [-0.31, -0.11]**

Heterogeneity: Tau² = 0.00; Chi² = 0.00, df = 1 (P = 0.95); I² = 0%
Test for overall effect: Z = 4.08 (P < 0.0001)

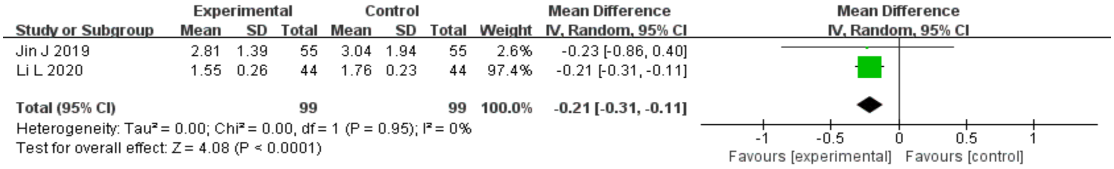

13. The results of sensitivity analysis of HDL-C for GQD combined with conventional treatment vs. conventional treatment.

Study omitted	Estimate	[95% Conf.	Interval]
Fu YH (2016)	.15238078	.1016103	.20315126
Wang L (2021)	.10687725	-.04490203	.25865653
Zhang J (2018)	.12207492	.05489228	.18925755
Combined	.12704195	.06497238	.18911151

14. The results of sensitivity analysis of LDL-C for GQD combined with conventional treatment vs. conventional treatment.

Study omitted	Estimate	[95% Conf.	Interval]
Chen FM (2022)	-.33240801	-.47566897	-.18914707
Fu YH (2016)	-.44185945	-.56266135	-.32105753
Gong J (2019)	-.39263323	-.5511263	-.23414016
Wang L (2021)	-.36605498	-.57327253	-.15883747
Xiong QJ (2019)	-.38654953	-.55125564	-.22184344
Zhang J (2018)	-.35465235	-.51254505	-.19675964
Zhou XY (2020)	-.39969575	-.55679435	-.24259721
Combined	-.38163764	-.52848845	-.23478684

15. The results of sensitivity analysis of LDL-C for GQD vs. conventional treatment.

Study omitted	Estimate	[95% Conf.	Interval]
Fan YF (2017)	-.2223997	-.40056911	-.04423028
Jin J (2019)	-.2521809	-.38597795	-.11838384
Li L (2020)	-.21059833	-.36490142	-.05629524
Combined	-.23142814	-.35576808	-.10708819

16. The results of sensitivity analysis of HOMA-IR for GQD combined with conventional treatment vs. conventional treatment.

Study omitted	Estimate	[95% Conf.	Interval]
Gong J (2019)	-.57896364	-1.0388548	-.11907241
Wang L (2021)	-1.5177823	-2.3748045	-.66076005
Wu L (2021)	-1.2145038	-1.9191941	-.50981349
Zhang LN (2019)	-1.6416659	-2.49314	-.79019213
Zhou XY (2020)	-1.7303667	-2.5650699	-.89566338
Combined	-1.1700027	-1.7920768	-.54792854

17. The results of sensitivity analysis of HOMA-IR for GQD vs. conventional treatment after changing the effect model.

Study or Subgroup	Experimental Mean	Experimental SD	Experimental Total	Control Mean	Control SD	Control Total	Weight	Std. Mean Difference IV, Fixed, 95% CI
Fan YF 2017	1.46	0.55	35	1.98	0.71	35	59.8%	-0.81 [-1.30, -0.32]
Li L 2020	38.74	4.92	44	51.17	3.76	44	40.2%	-2.81 [-3.41, -2.22]
**Total (95% CI)**			**79**			**79**	**100.0%**	**-1.61 [-1.99, -1.24]**

Heterogeneity: Chi² = 25.97, df = 1 (P < 0.00001); I² = 96%
Test for overall effect: Z = 8.37 (P < 0.00001)

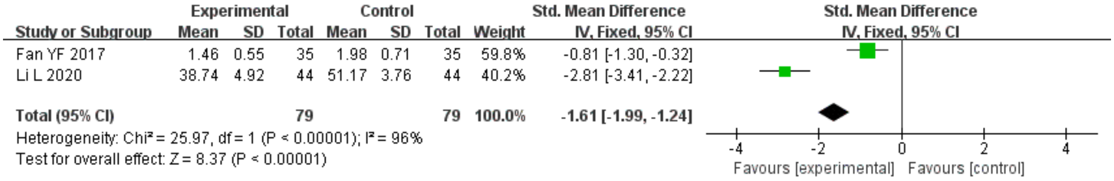

18. The results of sensitivity analysis of BMI for GQD combined with conventional treatment vs. conventional treatment after changing the effect model.

Study or Subgroup	Experimental Mean	Experimental SD	Experimental Total	Control Mean	Control SD	Control Total	Weight	Mean Difference IV, Random, 95% CI
Chen FM 2022	26.52	0.44	38	26.48	0.46	38	90.3%	0.04 [-0.16, 0.24]
Zhou XY 2020	26.59	1.65	45	26.71	1.33	45	9.7%	-0.12 [-0.74, 0.50]
**Total (95% CI)**			**83**			**83**	**100.0%**	**0.02 [-0.17, 0.22]**

Heterogeneity: Tau² = 0.00; Chi² = 0.23, df = 1 (P = 0.63); I² = 0%
Test for overall effect: Z = 0.25 (P = 0.80)

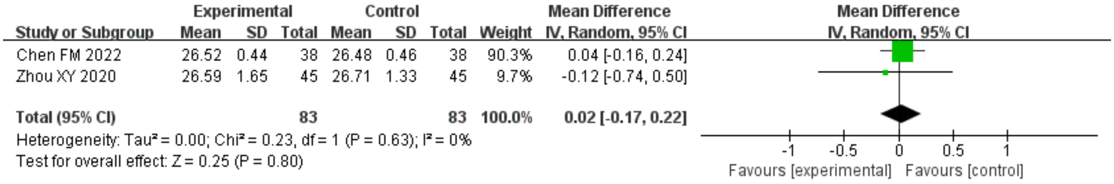

19. The results of sensitivity analysis of BMI for GQD vs. conventional treatment after changing the effect model.

Study or Subgroup	Experimental Mean	Experimental SD	Experimental Total	Control Mean	Control SD	Control Total	Weight	Mean Difference IV, Random, 95% CI
Fan YF 2017	26.53	1.56	35	26.94	1.8	35	62.1%	-0.41 [-1.20, 0.38]
Jin J 2019	27.75	3.73	55	27.33	2.04	55	37.9%	0.42 [-0.70, 1.54]
**Total (95% CI)**			**90**			**90**	**100.0%**	**-0.10 [-0.88, 0.69]**

Heterogeneity: Tau² = 0.10; Chi² = 1.40, df = 1 (P = 0.24); I² = 29%
Test for overall effect: Z = 0.24 (P = 0.81)

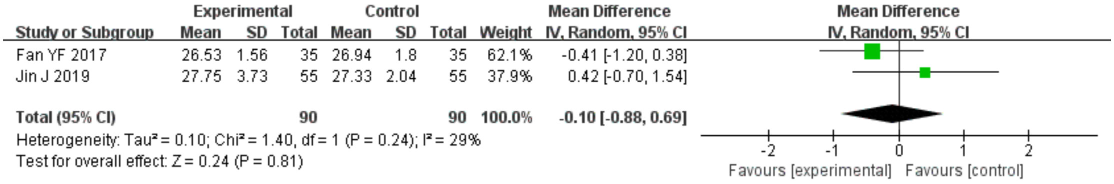

Summary of adverse reactions

Study	Sample Size (Randomized/ Analyzed) (E/C)	Gastrointestinal reactions (E/C)	Nephritis (E/C)	Headache (E/C)	Hypoglycemia (E/C))	Hypotension (E/C)	Electrocardiography (E/C)	Renal impairment (E/C)	Liver impairment (E/C)	Blood, urine and fecal routine (E/C)
Zhang LN,2019	172/172;86/86	0/0	0/0	0/0	0/0	0/0	0/0	0/0	0/0	0/0
Chen FM,2022	76/76;38/38	3/2	0/0	0/0	0/0	0/0	0/0	0/0	0/0	0/0
Fu YH2016	90/81;41/40	8/5	0/0	0/0	0/0	0/0	0/0	0/0	0/0	0/0
Wang QY,2021	80/80;40/40	2/1	2/1	1/0	0/0	0/1	0/0	1/0	0/0	0/0
Wang Y,2020	80/80;40/40	1/2	0/0	1/0	1/2	0/0	0/0	0/0	0/0	0/0
Wu L,2021	60/56;27/29	0/4	0/0	0/0	0/0	0/0	0/0	0/0	0/0	0/0
Zhong XF,2021	40/40;20/20	1/3	0/2	0/0	0/2	0/0	0/0	0/0	0/0	0/0
Zhou XY,2020	90/90;45/45	2/8	0/0	0/0	0/0	0/0	0/0	0/0	0/0	0/0
Fan YF,2017	70/70;35/35	5/1	0/0	0/0	0/0	0/0	0/0	0/0	0/0	0/0
Zhou A,2012	98/98;50/48	0/0	0/0	0/0	0/0	0/0	0/0	0/4	8/3	0/0

Egger's test of HBA1c, FBG and 2Hpg

1.Egger's test of FBG

Egger's test

Std_Eff	Coefficient	Std. err.	t	P>|t|	[95% conf.	interval]
slope	-.4650104	.1278547	-3.64	0.004	-.7464166	-.1836042
bias	-1.47967	.9643578	-1.53	0.153	-3.602207	.6428676

2. Egger's test of 2hPG

Egger's test

Std_Eff	Coefficient	Std. err.	t	P>|t|	[95% conf.	interval]
slope	-.916997	.2921688	-3.14	0.009	-1.560056	-.2739378
bias	-.1961112	1.02009	-0.19	0.851	-2.441314	2.049092

3. Egger's test of HbA1c

Egger's test

Std_Eff	Coefficient	Std. err.	t	P>|t|	[95% conf.	interval]
slope	-.8747294	.1392629	-6.28	0.000	-1.181245	-.5682138
bias	1.628402	1.21931	1.34	0.209	-1.05528	4.312085
